# Re-Infection Outcomes Following One- And Two-Stage Surgical Revision of Infected Knee Prosthesis: A Systematic Review and Meta-Analysis

**DOI:** 10.1371/journal.pone.0151537

**Published:** 2016-03-11

**Authors:** Setor K. Kunutsor, Michael R. Whitehouse, Erik Lenguerrand, Ashley W. Blom, Andrew D. Beswick, INFORM Team

**Affiliations:** Musculoskeletal Research Unit, School of Clinical Sciences, University of Bristol, Learning & Research Building (Level 1), Southmead Hospital, Southmead Road, Bristol, BS10 5NB, United Kingdom; Bern University of Applied Sciences, SWITZERLAND

## Abstract

**Background:**

Periprosthetic joint infection (PJI) is a serious complication of total knee arthroplasty. Two-stage revision is the most widely used technique and considered as the most effective for treating periprosthetic knee infection. The one-stage revision strategy is an emerging alternative option, however, its performance in comparison to the two-stage strategy is unclear. We therefore sought to ask if there was a difference in re-infection rates and other clinical outcomes when comparing the one-stage to the two-stage revision strategy.

**Objective:**

Our first objective was to compare re-infection (new and recurrent infections) rates for one- and two-stage revision surgery for periprosthetic knee infection. Our second objective was to compare between the two revision strategies, clinical outcomes as measured by postoperative Knee Society Knee score, Knee Society Function score, Hospital for Special Surgery knee score, WOMAC score, and range of motion.

**Design:**

Systematic review and meta-analysis.

**Data sources:**

MEDLINE, EMBASE, Web of Science, Cochrane Library, reference lists of relevant studies to August 2015, and correspondence with investigators.

**Study selection:**

Longitudinal (prospective or retrospective cohort) studies conducted in generally unselected patients with periprosthetic knee infection treated exclusively by one- or two-stage revision and with re-infection outcomes reported within two years of revision surgery. No clinical trials comparing both revision strategies were identified.

**Review methods:**

Two independent investigators extracted data and discrepancies were resolved by consensus with a third investigator. Re-infection rates from 10 one-stage studies (423 participants) and 108 two-stage studies (5,129 participants) were meta-analysed using random-effect models after arcsine transformation.

**Results:**

The rate (95% confidence intervals) of re-infection was 7.6% (3.4–13.1) in one-stage studies. The corresponding re-infection rate for two-stage revision was 8.8% (7.2–10.6). In subgroup analyses, re-infection rates remained generally similar for several study-level and clinically relevant characteristics. Postoperative clinical outcomes of knee scores and range of motion were similar for both revision strategies.

**Limitations:**

Potential bias owing to the limited number of one-stage revision studies and inability to explore heterogeneity in greater detail.

**Conclusions:**

Available evidence from aggregate published data suggest the one-stage revision strategy may be as effective as the two-stage revision strategy in treating infected knee prostheses in generally unselected patients. Further investigation is warranted.

**Systematic review registration:**

PROSPERO 2015: CRD42015017327

## Introduction

One of the most serious complications of total knee arthroplasty (TKA) is deep periprosthetic joint infection (PJI). Cure of PJI is not possible by antibiotic treatment alone in the majority of cases and surgical treatment is required. Options include: debridement, treatment with antibiotics and retention of the prosthesis; one-stage revision; two-stage revision; resection arthroplasty; or arthrodesis or amputation as a last resort [1, 2]. Most people will receive a one-stage or two-stage revision. The two-stage revision strategy has evolved as the gold standard and preferred procedure for treating PJI of the knee [3, 4]. However, this revision strategy is associated with long periods of hospitalization, lengthy functional impairment, and high health service costs [5]. For these reasons and though still less commonly used compared to the two-stage revision strategy, the one-stage strategy is an attractive alternative option. Besides the reduced number of surgical procedures for the patient, the one-stage procedure may be associated with a shorter overall time in hospital, reduced duration of postoperative antibiotic use, and quicker mobilization. It might also be a more cost-effective approach [5, 6]. The optimal treatment strategy for the management of deep prosthetic knee infection remains controversial.

To date, no randomized controlled trial has compared the effectiveness of the one- and two-stage revision procedures. However, a number of reviews based on observational evidence have assessed re-infection outcomes (recurrent and new infections) comparing both revision strategies and have mostly reported inconclusive results. In 2009, Jamsen and colleagues in their review of a total of 31 studies, reported overall infection eradication rates of between 73 to 100% and 82 to 100% in one- and two-stage revision strategies respectively [7]. In further assessment of the clinical outcomes as measured by knee scores and range of motion, there was no significant difference between the two strategies. In another review, Romano and colleagues reported average infection eradication rates of 89.8% and 81.8% in two-stage (38 studies) and one-stage (6 studies) revisions respectively and concluded that the two-stage was associated with a higher rate of infection eradication (albeit acknowledging the limitations posed by the methodological quality of included studies) [8]. In the most recent relevant review, Masters and colleagues reviewed available evidence (58 two-stage and 5 one-stage studies) and reported re-infection rates of between 0 to 41% for two-stage studies and 0 to 11% for one-stage studies [9]. In their report, the authors concluded that there was a significantly larger evidence base for the two-stage revision strategy and recommended further work to compare the two revision strategies.

The current evidence does not conclusively support a superior revision strategy for periprosthetic knee infection. In addition, several features of these previous reviews existed which limited the generalisability and validity of the findings. First, all of the reviews were characterised by a small number of one-stage revision studies (< 10 studies), which limited reliable comparability of the two revision strategies. Second, the heterogeneous periods of follow-up for re-infection outcomes in the individual studies were considered, which did not enhance comparability and interpretation of the findings. Third, none of the reviews explored for potential sources of heterogeneity among the contributing studies. Fourth, assessment of publication bias or small study bias was not conducted. Fifth, none of the reviews conducted any subgroup analysis across important study-level and clinically relevant characteristics (e.g. geographical location, age at baseline, and quality assessment). Sixth, apart from the study by Jamsen and colleagues [7] which was limited by its narrative approach, none of the studies compared the two revision strategies using other clinical outcomes. Finally, several reports have been published on the topic since the last previous relevant review [9].

In line with the uncertain evidence and recommendations of previous reviews [7, 9], there is a need for further work to compare the effectiveness of the two revision strategies. We therefore sought to ask if there was a difference in re-infection rates and other clinical outcomes when comparing the one-stage to the two-stage revision strategy. Ideally, to compare the effectiveness of these two revision strategies will require evidence from a carefully designed randomized clinical trial; however, this is may be unlikely in the short term given the low PJI event rates recorded after TKA [10, 11]. In the absence of robust evidence from a rigorous randomized clinical trial; using a systematic meta-analytic approach, our aim was to (1) to evaluate the effectiveness of one- and two-stages revision strategies using re-infection rate (primary outcome) and other clinical outcomes as measured by postoperative Knee Society Knee score, Knee Society Function score, Hospital for Special Surgery knee score, WOMAC score, and range of motion following revision arthroplasty and (2) to compare and describe the outcomes between the two revision strategies.

## Methods

### Data sources and search strategy

Our review has been registered in the PROSPERO prospective register of systematic reviews (CRD42015017327) and was conducted using a predefined protocol and in line with PRISMA (Appendix A in S1 File) and MOOSE guidelines [12, 13] (Appendix B in S1 File). We searched for longitudinal studies (retrospective, prospective, or randomized controlled trials) reporting re-infection outcomes following one- or two-stage surgical revision of infected knee prosthesis in MEDLINE, EMBASE, Web of Science, and Cochrane databases from inception up to August 2015. The search strategy used a combination of key words related to knee replacement, infection, and revision with focus on one- and two-stage surgeries. No language restrictions were employed. We complemented the search by manually scanning reference lists of identified articles and review articles for relevant publications missed by the original search. Details on our search strategy are presented in Appendix C in S1 File.

### Eligibility criteria

We included studies comprising of consecutive generally unselected patients (i.e., patients representative of the general patient population) who were treated exclusively by one-stage or two-stage revision with a follow-up duration of at least two years for re-infection outcomes (recurrent and new infections). We excluded: (i) studies that reported case series of methods in selected group of patients (such as patients with a specific infection); (ii) studies that did not include patients with less than two years of follow-up; and (iii) studies with less than 10 participants as these were more likely to be case series which did not include consecutive patients.

### Data extraction and quality assessment

After an initial screen of abstracts by one reviewer (S.K.K.), potentially relevant articles were acquired. Each article was assessed by two independent reviewers (S.K.K., A.D.B.) using the inclusion criteria and any discrepancies regarding eligibility of an article was discussed, and consensus reached with a third author (M.R.W). One author (S.K.K.) independently extracted data and performed quality assessments using a standardized data collection form. A second reviewer checked data with that in the original articles. Data were extracted on study design, year of publication, geographical location, mean baseline age, proportion of males, period of follow-up following revision surgery, type of fixation used for re-implantation used, use of and type of spacer, number of re-infection outcomes and participants, and clinical characteristics such as preoperative and postoperative Knee Society Knee score, Knee Society Function score, Hospital for Special Surgery knee score, WOMAC score, and range of motion. For multiple publications involving the same study, the most comprehensive study was used. We corresponded with study investigators to provide missing information. Information on the methodological quality of included studies was assessed based on the Methodological Index for Non-Randomised Studies (MINORS), a validated instrument which is designed for assessment of methodological quality of non-randomized studies in surgery [14] and has been described previously.[15] Briefly, it uses eight pre-defined domains namely: a clearly stated aim, inclusion of consecutive patients, prospective collection of data, endpoints appropriate to the aim of the study, unbiased assessment of the study endpoint, follow-up period appropriate to the aim of the study, loss to follow-up less than 5%, and prospective calculation of the study size. For non-comparative studies, the score varies from 0 to 16, which is the global ideal score.

### Statistical analysis

The rate of re-infection (estimated from the number of re-infections within two years of knee revision surgery/total number of participants or arthroplasties as reported) with 95% confidence intervals (CIs) was used as the summary measure and primary outcome across studies. The Freeman-Tukey variance stabilising double arcsine transformation [16] was used in estimating the rates, because the alternative use of inverse variance weight in fixed-effects meta-analysis is suboptimal when dealing with binary data with low rates. Details of the method have been reported previously [15]. To account for the effect of between-study heterogeneity anticipated, summary rates of re-infection were pooled using random effects models [17]. We also estimated 95% prediction intervals which are used to determine the degree of heterogeneity, as they provide a region in which about 95% of the true study effects are expected to be found [18, 19]. Standard chi-square tests and the I^2^ statistic [20] were used to quantify the extent of statistical heterogeneity across studies. We explored for potential sources of heterogeneity, including study-level and clinically relevant characteristics such as geographical location, baseline mean age, use of a spacer and spacer type, type of fixation used in reimplantation (cemented or cementless), size of study, and study quality, using stratified analysis and random effects meta-regression [21, 22]. We assessed for publication bias using Egger’s regression symmetry test [23]. In the presence of publication bias, we adjusted for this using the Duval and Tweedie’s nonparametric trim-and-fill method [24]. Though the trim-and-fill method provides fairly conservative estimates compared to the less commonly used Copas selection model [25], we employed the trim-and-fill method as it is commonly used in meta-analysis of this nature and the Copas selection model is also cumbersome to employ and frequently encounters statistical estimation. Nonetheless, the two methods give similar point estimates [25]. Given the suggested inefficiency of Begg’s funnel plots as a method for assessing publication bias in meta-analysis of proportion studies with low proportion outcomes [26], this was not employed. Due to the unsuitability of pooling data for the clinical outcomes of postoperative Knee Society Knee score, Knee Society Function score, Hospital for Special Surgery knee score, WOMAC score, and range of motion, these outcomes were compared between the two revision strategies using descriptive statistics. We employed Stata version 14 (Stata Corp, College Station, Texas, USA) for all statistical analyses. The complete dataset used for our analyses can be found in S2 File.

## Results

### Study identification and selection

**Fig 1:**
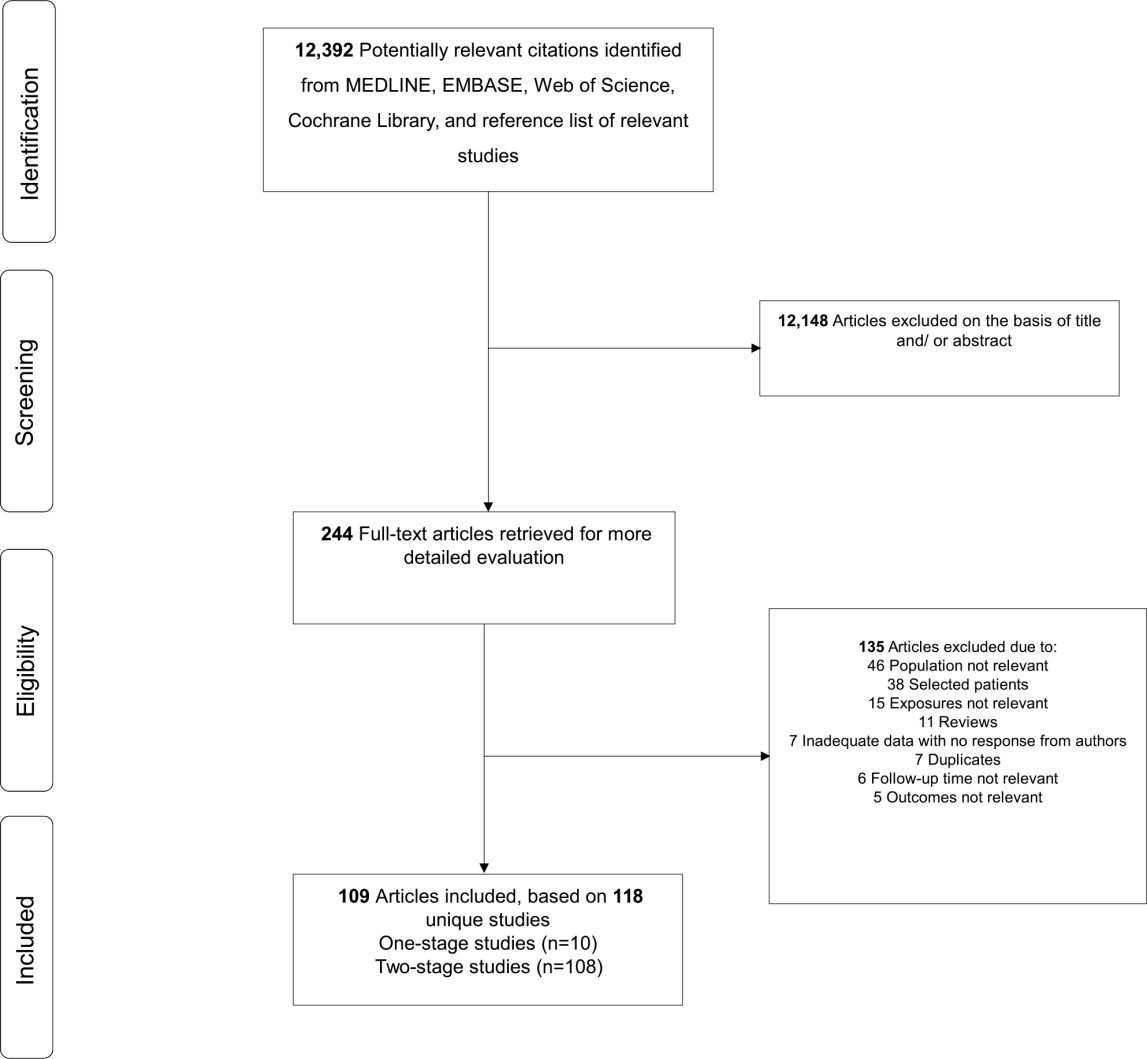
PRISMA Flow Diagram.

Our computer search and manual screening of reference lists of relevant studies identified 12,392 potentially relevant citations. After initial screening based of titles and abstracts, 244 articles remained for further evaluation. We excluded 135 articles following detailed assessments (Appendix D in S1 File). The remaining 109 articles were relevant to the review question and were included in the meta-analysis (Fig 1; Table 1; Table A and Appendix E in S1 File). Overall, there were 118 independent studies (comprising of 5,552 participants and 667 re-infection outcomes) included in the review.

**Table 1:** Summary Characteristics of Included Studies.

	One-stage revision	Two-stage revision
**Eligible studies**		
** Total number of studies included**	10	108
**Participants**		
** Total number of re-infections**	42	625
** Total number of participants**	423	5,129
** Median (IQR) age (years)**	71.1 (66.8–71.8)	67.0 (65.2–70.0)
**Location**		
** Europe**	9 (401)	36 (1,181)
** North America**	1 (22)	52 (3,468)
** Asia**	-	18 (448)
** Pacific**	-	2 (32)
**Study characteristics**		
** Median (IQR) follow-up (months)**	52.0 (24.0–122.4)	47.0 (32.2–62.2)
** Cemented reimplants**	3 (97)	38 (1,349)
** Use of spacers**	-	88 (4,082)
** Interval between stages (months)**	-	3.2 (2.0–4.7)
**Baseline clinical characteristics**		
** Median (IQR) Knee Society knee score**	48.0 (32.0–55.0)	45.8 (35.1–64.0)
** Median (IQR) Knee Society function score**	33.0 (24.0–42.0)	28.3 (16.6–37.4)
Median (IQR) Hospital for Special Surgery knee score*	-	48.2 (42.4–57.6)
Median (IQR) WOMAC score*	-	49.5 (45.7–60.7)
** Median (IQR) range of motion (degrees)**	84.5 (79.0–90.0)	74.1 (64.0–83.0)

IQR = interquartile range; values are number of studies (number of participants) unless stated otherwise

*, none of the one-stage revision strategy studies reported baseline scores for these clinical characteristics

### Study characteristics and study quality

Table 1 provides summary characteristics of one- and two-stage studies included in the review. Table A in S1 File provides baseline characteristics and quality assessment scores of the individual studies. All studies were longitudinal (prospective or retrospective cohort) studies carried out in Europe (United Kingdom, Italy, Germany, France, Switzerland, Austria, Sweden, Spain, Italy, Finland, Greece, Poland, Netherlands, Denmark, Czech Republic, Turkey, and Belgium), North America (United States of America and Canada), Asia (South Korea, India, China, and Taiwan), and the Pacific (Australia and New Zealand). No clinical trials comparing both revision strategies were identified. The methodological quality of included studies ranged from 9–15.

### One-stage revision

**Fig 2:**
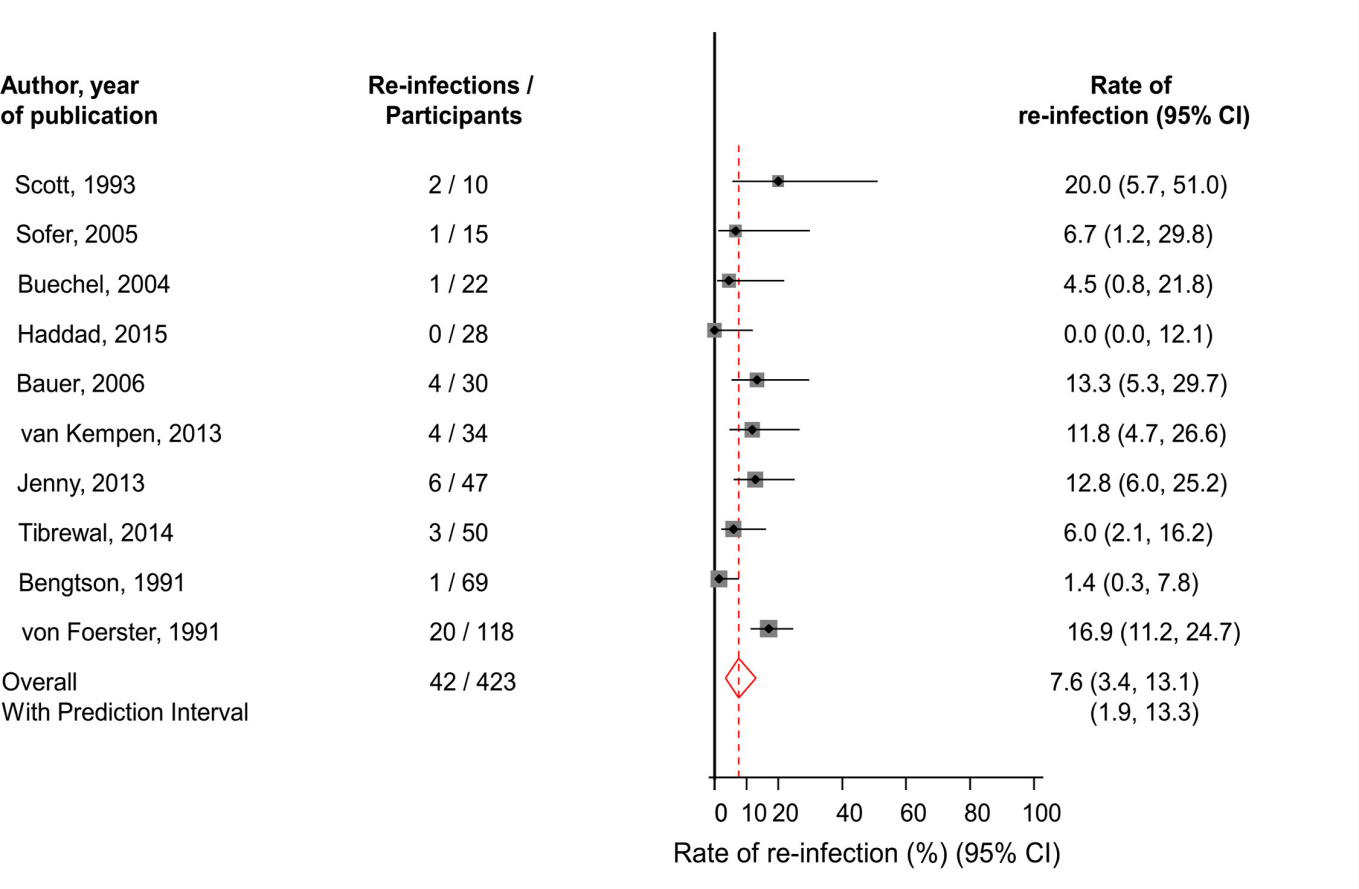
Rates of Re-Infection in Patients Treated by One-Stage Revision. The summary estimates presented were calculated using random effects models; CI, confidence interval (bars)

Ten studies comprising of 423 participants (42 re-infections) reported on re-infection outcomes in patients using the one-stage surgical revision strategy (Table 1; Table A in S1 File). The pooled random effects re-infection rate (95% CI) was 7.6% (3.4–13.1; *P* < 0.001) (Fig 2). The 95% prediction interval for the summary re-infection rate was 1.9 to 13.3%, suggesting that the true re-infection rate for any single study will usually fall within this range. The moderate heterogeneity between contributing studies (*I*^*2*^ = 62%, 95% CI: 24–81%; *P* = 0.005) was not explained by any study-level characteristics explored (*P* for meta-regression > 0.10 for each; Figure A in S1 File). The rates of re-infection did not vary significantly by levels or categories of several study-level characteristics. Heterogeneity was substantially reduced when we restricted analyses to studies of the highest quality (quality score: ≥ 12) (*I*^*2*^ = 42%, 95% CI: 0–79%; *P* = 0.144). Among the higher quality studies, the pooled re-infection rate (95% CI) was 6.4% (2.1–12.4; *P* < 0.001), which was similar to the main finding. In further exploration of heterogeneity, on exclusion of the single largest study comprising of 118 participants (with 20 re-infections) [27], heterogeneity was substantially reduced (*I*^*2*^ = 48%, 95% CI: 0–76%; *P* = 0.053) and the pooled re-infection rate (95% CI) was 6.2% (2.4–11.2; *P* < 0.001). There was no statistically significant evidence of publication bias using the Egger test (*P* = 0.516), consistent with the absence of selective reporting when studies were grouped by size (Figure A in S1 File).

### Two-stage revision

**Fig 3:**
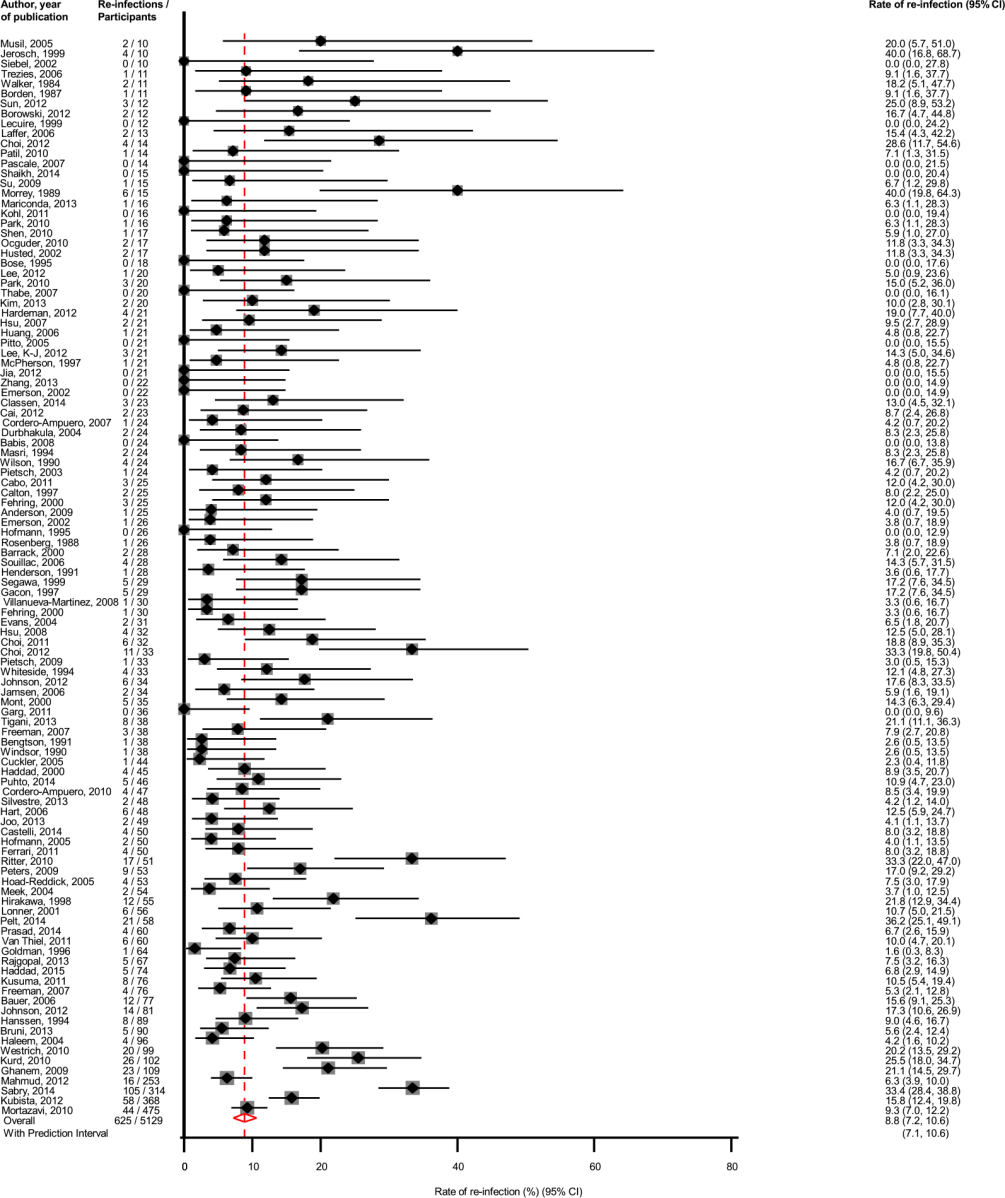
Rates of Re-Infection in Patients Treated by Two-Stage Revision. The summary estimates presented were calculated using random effects models; CI, confidence interval (bars)

Assessment of re-infection outcomes using the two-stage surgical revision strategy was reported in 108 studies involving 5,129 participants and 625 re-infection outcomes (Table 1; Table A in S1 File). The pooled re-infection rate (95% CI) was 8.8% (7.2–10.6; *P* < 0.001) (Fig 3). The corresponding 95% prediction interval was 7.1 to 10.6%, similar to the 95% confidence interval. There was evidence of substantial heterogeneity between contributing studies (*I*^*2*^ = 71%, 64–76%; *P* < 0.001), which was unexplained by any of the study-level characteristics assessed (Figure B in S1 File). On simultaneous exclusion of the two largest studies [28, 29], the pooled re-infection rate (95% CI) was 8.6% (7.1–10.2; *P* < 0.001), which was similar to the main finding. The rates of re-infection were generally similar across study relevant characteristics. There was no evidence of heterogeneity (*I*^*2*^ = 0%, 0–68%; *P* = 0.602), when we restricted the analysis to studies of the highest quality. Among the higher quality studies, the pooled re-infection rate (95% CI) was 6.1 (3.3–9.2; *P* < 0.001).There was evidence of publication bias (Egger’s *P* = 0.008) and Duval and Tweedie’s trim-and-fill method resulted in no additional imputed studies (Figure C in S1 File).

### Other clinical outcomes

Clinical outcomes as measured by postoperative Knee Society Knee score, Knee Society Function score, and range of motion following revision arthroplasty, were similar between both revision strategies (Table 2); however, only a limited number of one-stage studies reported scores for these outcomes.

**Table 2:** Post-Operative Clinical Outcomes Following One- and Two-Stage Revision Strategies.

	One-stage revision	Two-stage revision
**Median (IQR) Knee Society knee score**	80.3 (74.8–86.5)	82.1 (76.0–86.0)
**Median (IQR) Knee Society function score**	62.5 (61.0–78.0)	68.0 (59.4–77.9)
Median (IQR) Hospital for Special Surgery knee score*	-	82.5 (79.2–87.0)
Median (IQR) WOMAC score*	-	45.5 (30.3–66.4)
**Median (IQR) range of motion (degrees)**	97.5 (93.8–100.5)	97.8 (93.7–104.0)

IQR = interquartile range

*, none of the one-stage revision strategy studies reported post-operative scores for these clinical outcomes

## Discussion

### Key findings

Given the uncertainty regarding the performance of the one-stage revision strategy in comparison to the two-stage strategy when treating periprosthetic knee infection, we sought to ask if there was a difference in re-infection rates and other clinical outcomes when comparing the one-stage to the two-stage revision strategy. Using a systematic and meta-analytical approach, we aimed to compare the effectiveness of the one- and two-stages revision strategies, using re-infection rate as a primary outcome and other clinical outcomes as measured by postoperative Knee Society Knee score, Knee Society Function score, Hospital for Special Surgery knee score, WOMAC score, and range of motion following revision arthroplasty. We found similar re-infection rates following one- or two-stage surgical revision for infected knee prostheses. For both revision strategies, our findings also showed that re-infection rates were generally similar (albeit with overlapping confidence intervals) across several study relevant characteristics. The findings for other clinical outcomes were similar for both revision strategies; however, in the context of the limited data for one-stage revision studies, these findings should be interpreted with caution

### Comparison with previous work

Some of our findings are consistent with that of previous reviews on the topic. We also provide several findings that have not been previously reported. Our findings suggest the one-stage as an equivalent revision strategy to the two-stage in terms of effectiveness for treating periprosthetic knee infection, consistent with the results of some previous reviews [7, 9]. However, in contrast to the narrative approach used by Masters et al.[9] and Jamsen et al.[7], we employed a meta-analytic approach, thereby providing more specific and reliable estimates of re-infection rates after one- and two-stage revision of infected knee prosthesis. Our results were also based on a larger number of studies (108 two-stage and 10 one-stage studies), therefore we had enhanced power to robustly demonstrate these results. For the first time, we have also shown that re-infection rates were generally similar across several study-level characteristics such as geographical location, mean baseline age, size of study, and study quality for both revision strategies. Finally, pooled estimates reported from available studies contributing data, showed that the effectiveness of the two revision strategies was similar as measured by other clinical outcomes such as knee scores and range of motion.

### Implications of our findings

Given that the control of infection and maintenance of joint function are considered as main criteria for a successful outcome following one- or two stage revision [6, 30], our findings suggest that the one-stage revision strategy may be as effective as the two-stage strategy in treating many patients with periprosthetic knee infection. The two-stage revision strategy which was pioneered and described by Insall and colleagues in 1983 [31], has for several decades been the established gold standard for treating infected knee replacements [4, 32]. Despite the high success rate in eradication of infection, the two-stage strategy frequently results in significant functional impairment and occasionally death [33]. It is also a costly process in terms of both time and money. Surgical revision of a total knee replacement has been estimated to consume considerably more resources than a primary total knee replacement [34, 35], with even more resources required for surgical revision of an infected arthroplasty [36]. The average cost to the NHS of surgical revision of an infected knee replacement is estimated to be about £30 011, which is three times more than that of an aseptic revision [37]. With increasing life expectancy, a growing healthcare burden due to osteoarthritis [38], and a predicted large rise in the numbers of primary TKA being performed, there will be a proportionate rise in the number of patients requiring revision surgery for infected knee prostheses in the coming decades [39, 40]. Within the NHS, total knee arthroplasty is one of the most common orthopaedic procedures [41]. Given the current economic climate and the financial burden associated with the two-stage revision strategy, optimisation of limited resources is of crucial importance.

With the establishment in the treatment of hip periprosthetic joint infection of the one-stage revision strategy by Buchholz and colleagues over four decades ago [42], its use has become increasingly popular in recent years. The one-stage revision is widely used in infected hip arthroplasties, but has not gained the same level of momentum in the knee. However, there is an increasing enthusiasm for this procedure in some centres [6] and has been adopted in a number of small case series [43–45]. Apart from the economic benefits associated with this procedure, it has been reported that the one-stage strategy may be associated with better maintenance of joint function (allowing earlier mobility) in selected cases [5]. However, comparing the one-stage procedure in knee and hips, less favourable results have been reported in the knee [1, 46]. One-stage surgical revision has traditionally been thought to expose the patient to a higher risk of re-infection by any residual bacteria [47], given the limited opportunities for additional antimicrobial strategies associated with it. Therefore, suggestions have been made that this strategy should be used only in selected cases, such as patients with minimal bone loss, known organisms with known sensitivities, absence of a sinus tract, and non-immunocompromised patients [48, 49]. Our findings thus yield supportive evidence for the one-stage strategy as a potential alternative option among unselected patients in general.

### Strengths and limitations

The strengths of the current review deserve mention. First, given the large number of studies included (twice as many one- and two-stage studies than the most recent previous review [9]), we had enhanced power to reliably compare the effectiveness of one- and two-stage revision strategies in more detail. Second, we conducted a detailed assessment of the methodological quality of the included studies using a validated instrument for non-randomised surgical studies. Third, compared to previous reviews [7, 9], we employed a meta-analytic approach, thereby the ability to compare the effectiveness of the two revision strategies using quantitative estimates. Fourth, we compared re-infection rates among a broad range of study-level and clinically relevant characteristics. Fifth, we also compared the effectiveness of the two revision strategies using post-operative clinical outcomes such as knee scores and range of motion. Sixth, our results remained robust in several sensitivity analyses. Indeed, our confidence and estimated prediction intervals were similar. Finally, we quantified heterogeneity and explored for potential sources of bias, which included heterogeneity and small study effects.

The limitations also deserve consideration. We acknowledge the limited number of one-stage revision studies, which precluded the ability to robustly compare re-infection rates and other clinical outcomes between the two interventions. We could not conduct detailed subgroup analyses by relevant subgroups such as co-morbidities (e.g., history of diabetes) and duration of antibiotic therapy, given the limited data. We aimed to include only studies that that did not involve selected patients, however, this may not have been possible in some instances given the earlier phase of selection related to management without further replacement in some studies. Though we aimed to include studies that reported re-infection outcomes within two years of revision surgery, there is a possibility some re-infection outcomes may have occurred outside this period as some authors did not respond to requests for further information. Our findings are comprehensive, important and timely, but should be interpreted with caution in the context of the level of evidence. Indeed, to robustly compare the effectiveness of one-stage and two-stage revision strategies, will require evidence from a carefully designed randomized clinical trial.

## Conclusions

Available evidence from aggregate published data suggest the one-stage revision strategy may be as effective as the two-stage revision strategy in treating infected knee prostheses in generally unselected patients. Further investigation is warranted.

## Supporting Information

S1 File (DOC)

S2 File (CSV)
