# Supplementary material for: Re-Infection Outcomes Following One- And Two-Stage Surgical Revision of Infected Knee Prosthesis: A Systematic Review and Meta-Analysis
Source: PLoS One. 2016 Mar 11;11(3):e0151537. doi: 10.1371/journal.pone.0151537 (PMC4788419; doi:10.1371/journal.pone.0151537)
Supplement: S1 File — (DOC) [file pone.0151537.s001.doc]

**S1 File**

| **Appendix A** | PRISMA checklist |
| --- | --- |
| **Appendix B** | MOOSE checklist |
| **Appendix C** | Literature search strategy |
| **Appendix D** | Reference list of excluded studies |
| **Appendix E** | Reference list of studies included in review |
| **Table A** | Characteristics of prospective studies included in review |
| **Figure A** | Rates of re-infection in patients treated by one-stage revision, grouped according to study and population level characteristics |
| **Figure B** | Rates of re-infection in patients treated by two-stage revision, grouped according to study and population level characteristics |
| **Figure C** | Filled funnel plot with 95% confidence limits after trim-and-fill method |

**Appendix A.** PRISMA checklist

| **Section/topic** | **Item No** | **Checklist item** | **Reported on page No** |
| --- | --- | --- | --- |
| **Title** | | | |
| Title | 1 | Identify the report as a systematic review, meta-analysis, or both | 1 |
| **Abstract** | | | |
| Structured summary | 2 | Provide a structured summary including, as applicable, background, objectives, data sources, study eligibility criteria, participants, interventions, study appraisal and synthesis methods, results, limitations, conclusions and implications of key findings, systematic review registration number | 2 |
| **Introduction** | | | |
| Rationale | 3 | Describe the rationale for the review in the context of what is already known | 4-5 |
| Objectives | 4 | Provide an explicit statement of questions being addressed with reference to participants, interventions, comparisons, outcomes, and study design (PICOS) | 5 |
| **Methods** | | | |
| Protocol and registration | 5 | Indicate if a review protocol exists, if and where it can be accessed (such as web address), and, if available, provide registration information including registration number | 2 |
| Eligibility criteria | 6 | Specify study characteristics (such as PICOS, length of follow-up) and report characteristics (such as years considered, language, publication status) used as criteria for eligibility, giving rationale | 6 |
| Information sources | 7 | Describe all information sources (such as databases with dates of coverage, contact with study authors to identify additional studies) in the search and date last searched | 6 |
| Search | 8 | Present full electronic search strategy for at least one database, including any limits used, such that it could be repeated | Appendix C |
| Study selection | 9 | State the process for selecting studies (that is, screening, eligibility, included in systematic review, and, if applicable, included in the meta-analysis) | 6-7 |
| Data collection process | 10 | Describe method of data extraction from reports (such as piloted forms, independently, in duplicate) and any processes for obtaining and confirming data from investigators | 6-7 |
| Data items | 11 | List and define all variables for which data were sought (such as PICOS, funding sources) and any assumptions and simplifications made | 6-7 |
| Risk of bias in individual studies | 12 | Describe methods used for assessing risk of bias of individual studies (including specification of whether this was done at the study or outcome level), and how this information is to be used in any data synthesis | 7-8 |
| Summary measures | 13 | State the principal summary measures (such as risk ratio, difference in means). | 7-8 |
| Synthesis of results | 14 | Describe the methods of handling data and combining results of studies, if done, including measures of consistency (such as I^2^ statistic) for each meta-analysis | 7-8 |
| Risk of bias across studies | 15 | Specify any assessment of risk of bias that may affect the cumulative evidence (such as publication bias, selective reporting within studies) | 7-8 |
| Additional analyses | 16 | Describe methods of additional analyses (such as sensitivity or subgroup analyses, meta-regression), if done, indicating which were pre-specified | 7-8 |
| **Results** | | | |
| Study selection | 17 | Give numbers of studies screened, assessed for eligibility, and included in the review, with reasons for exclusions at each stage, ideally with a flow diagram | 8 and Fig. 1 |
| Study characteristics | 18 | For each study, present characteristics for which data were extracted (such as study size, PICOS, follow-up period) and provide the citations | 8-9, Table A |
| Risk of bias within studies | 19 | Present data on risk of bias of each study and, if available, any outcome-level assessment (see item 12). | 9-10, Table A |
| Results of individual studies | 20 | For all outcomes considered (benefits or harms), present for each study (a) simple summary data for each intervention group and (b) effect estimates and confidence intervals, ideally with a forest plot | 9-10, Figs. 2-3 |
| Synthesis of results | 21 | Present results of each meta-analysis done, including confidence intervals and measures of consistency | 9-10, Figs. 2-3 |
| Risk of bias across studies | 22 | Present results of any assessment of risk of bias across studies (see item 15) | 9-10 |
| Additional analysis | 23 | Give results of additional analyses, if done (such as sensitivity or subgroup analyses, meta-regression) (see item 16) | 9-10, Figures A and B |
| **Discussion** | | | |
| Summary of evidence | 24 | Summarise the main findings including the strength of evidence for each main outcome; consider their relevance to key groups (such as health care providers, users, and policy makers) | 10-11 |
| Limitations | 25 | Discuss limitations at study and outcome level (such as risk of bias), and at review level (such as incomplete retrieval of identified research, reporting bias) | 11-12 |
| Conclusions | 26 | Provide a general interpretation of the results in the context of other evidence, and implications for future research | 12 |
| **Funding** | | | |
| Funding | 27 | Describe sources of funding for the systematic review and other support (such as supply of data) and role of funders for the systematic review | 13 |

**Appendix B.** MOOSE checklist

**Re-Infection Outcomes Following One- and Two-Stage Surgical Revision of Infected Knee Prosthesis: A Systematic Review and Meta-Analysis**

| **Criteria** | | **Brief description of how the criteria were handled in the review** |
| --- | --- | --- |
| **Reporting of background** | |  |
| √ | Problem definition | Deep prosthetic joint infection is a dreaded complication of total knee arthroplasty. Two main treatment options exist and which include one-stage or two-stage revision. Several studies and reviews have been carried out on this topic, but the best treatment option is currently uncertain. In this context, we have carried out an updated systematic review and meta-analysis of longitudinal studies to compare the effectiveness of the one- and two-stage revision strategies in terms of re-infection outcomes |
| √ | Hypothesis statement | There is no difference in re-infection outcomes after one-stage or two-stage revision surgery for prosthetic knee infection |
| √ | Description of study outcomes | Rates of re-infection (number of re-infections and or recurrence of infection within two years of knee revision surgery/total number of participants); Postoperative Knee Society Knee Score; Knee Society Function Score; Hospital for Special Surgery knee score; WOMAC score; and range of motion. |
| √ | Type of exposure | One-stage and two-stage surgical revision of infected knee prosthesis |
| √ | Type of study designs used | Longitudinal studies (retrospective, prospective, or randomised controlled trials) |
| √ | Study population | Generally unselected patients (i.e., patients representative of the general patient population) treated exclusively by one-stage or two-stage revision |
| **Reporting of search strategy should include** | |  |
| √ | Qualifications of searchers | Setor Kunutsor, PhD; Andrew Beswick, BSc |
| √ | Search strategy, including time period included in the synthesis and keywords | Time period: From inception to August, 2015.  The detailed search strategy can be found in Appendix 3. |
| √ | Databases and registries searched | MEDLINE, EMBASE, Web of Science, and Cochrane databases |
| √ | Search software used, name and version, including special features | OvidSP was used to search EMBASE and MEDLINE  EndNote used to manage references |
| √ | Use of hand searching | We searched bibliographies of retrieved papers |
| √ | List of citations located and those excluded, including justifications | Details of the literature search process are outlined in the flow chart. The citation list for excluded studies is in Appendix 5. |
| √ | Method of addressing articles published in languages other than English | We placed no restrictions on language |
| √ | Method of handling abstracts and unpublished studies | We contacted several investigators for unpublished data and abstracts on the associations |
| √ | Description of any contact with authors | We contacted authors of studies that did not provide adequate data for analysis |
| **Reporting of methods should include** | |  |
| √ | Description of relevance or appropriateness of studies assembled for assessing the hypothesis to be tested | Detailed inclusion and exclusion criteria are described in the Methods section. |
| √ | Rationale for the selection and coding of data | Data extracted from each of the studies were relevant to the population characteristics, study design, exposure, and outcome. |
| √ | Assessment of confounding | We included only studies where populations were unselected |
| √ | Assessment of study quality, including blinding of quality assessors; stratification or regression on possible predictors of study results | Study quality was assessed based on the Methodological Index for Non-Randomised Studies (MINORS), a validated instrument which is designed for assessment of methodological quality of non-randomised studies in surgery |
| √ | Assessment of heterogeneity | Heterogeneity of the studies was quantified with I^2^ statistic that provides the relative amount of variance of the summary effect due to the between-study heterogeneity and explored using meta-regression and stratified analyses |
| √ | Description of statistical methods in sufficient detail to be replicated | Description of methods of meta-analyses, sensitivity analyses, meta-regression and assessment of publication bias are detailed in the methods. We performed random effects meta-analysis with Stata 14. |
| √ | Provision of appropriate tables and graphics | Tables 1-2 and Table A; Figs. 2 and 3; Figures A and B |
| **Reporting of results should include** | |  |
| √ | Graph summarizing individual study estimates and overall estimate | Figs. 2-3 |
| √ | Table giving descriptive information for each study included | Table 1 and Table A |
| √ | Results of sensitivity testing | Sensitivity analysis was conducted to assess the influence of some large studies and low quality studies on the pooled estimate. This was done by omitting such studies and calculating a pooled estimate for the remainder of the studies |
| √ | Indication of statistical uncertainty of findings | 95% confidence intervals were presented with all summary estimates, I^2^ values and results of sensitivity analyses |
| **Reporting of discussion should include** | |  |
| √ | Quantitative assessment of bias | Sensitivity analyses indicate heterogeneity in strengths of the association due to most common biases in observational studies. The systematic review is limited in scope, as it involves published data. Individual participant data is needed. Limitations have been discussed. |
| √ | Justification for exclusion | All studies were excluded based on the pre-defined inclusion criteria in methods section. |
| √ | Assessment of quality of included studies | Brief discussion included in ‘Methods’ section |
| **Reporting of conclusions should include** | |  |
| √ | Consideration of alternative explanations for observed results | Discussion |
| √ | Generalization of the conclusions | Discussed in the context of the results. |
| √ | Guidelines for future research | We recommend evidence from a carefully designed randomised clinical trial |
| √ | Disclosure of funding source | In “Source of Funding” section |

**Appendix C.** Literature search strategy

Relevant studies, published before August 31, 2015 (date last searched), were identified through electronic searches not limited to the English language using MEDLINE, EMBASE, Web of Science, and Cochrane databases. Electronic searches were supplemented by scanning reference lists of articles identified for all relevant studies (including review articles), by hand searching of relevant journals and by correspondence with study investigators. The computer-based searches combined search terms related to hip replacement, infection, and revision with focus on one- and two stage surgeries without language restriction.

1 exp Prosthesis-Related Infections/ or prosthesis-related infection*.mp. (8340)

2 exp Infection/ or infection.mp. (1209021)

3 wound infection.mp. or exp Wound Infection/ (44719)

4 exp Surgical Wound Infection/ or surgical infection.mp. (29431)

5 exp Sepsis/ or sepsis.mp. (129398)

6 1-stage.mp. (1578)

7 2-stage.mp. (2833)

8 one stage.mp. (8710)

9 two stage.mp. (15993)

10 one-stage.mp. (8710)

11 two-stage.mp. (15993)

12 single stage.mp. (4354)

13 single-stage.mp. (4354)

14 exchange.mp. (231326)

15 exp Prosthesis-Related Infections/ or direct exchange.mp. (8465)

16 direct-exchange.mp. (153)

17 revision arthroplasty.mp. or exp Knee Prosthesis/ (9737)

18 staged revision.mp. (41)

19 reoperation.mp. or exp Reoperation/ (77363)

20 reimplantation.mp. or exp Replantation/ (10457)

21 reimplant*.mp. (6348)

22 1 or 2 or 3 or 4 or 5 (1235959)

23 6 or 7 or 8 or 9 or 10 or 11 or 12 or 13 or 14 or 15 or 16 or 17 or 18 or 19 or 20 or 21 (359475)

24 arthroplasty.mp. or exp Arthroplasty, Replacement, Knee/ or exp Arthroplasty, Replacement/ or exp Arthroplasty/ (52051)

25 exp Arthroplasty, Replacement/ or Replacement.mp. or exp Arthroplasty, Replacement, Knee/ (204563)

26 exp Knee/ or exp Arthroplasty, Replacement, Knee/ or knee.mp. (105895)

27 exp Knee Joint/ or exp Arthroplasty, Replacement, Knee/ or exp Joint Prosthesis/ or knee replacement.mp. or exp Knee Prosthesis/ (83294)

28 exp Joint Prosthesis/ or exp Knee Prosthesis/ or exp Arthroplasty, Replacement, Knee/ or Total knee.mp. or exp Knee Joint/ (83497)

29 Knee arthroplasty.mp. or exp Arthroplasty, Replacement, Knee/ (16575)

30 Knee prosthesis.mp. or exp Knee Prosthesis/ (9392)

31 Total knee replacement.mp. or exp Arthroplasty, Replacement, Knee/ (14877)

32 exp Arthroplasty/ or exp Knee Joint/ or exp Arthroplasty, Replacement, Knee/ or Total knee arthroplasty.mp. or exp Knee Prosthesis/ (83930)

33 24 or 25 or 26 or 27 or 28 or 29 or 30 or 31 or 32 (312674)

34 22 and 23 and 33 (7306)

35 limit 34 to humans (7197)

Each part was specifically translated for searching the other databases (EMBASE, Web of Science, and Cochrane databases)

**Appendix D.** Reference list of excluded studies

1. Achermann Y, Stasch P, Preiss S, Lucke K, Vogt M. Characteristics and treatment outcomes of 69 cases with early prosthetic joint infections of the hip and knee. *Infection.* 2014;42(3):511-519.

2. Watts CD, Wagner ER, Houdek MT, et al. Morbid obesity: a significant risk factor for failure of two-stage revision total knee arthroplasty for infection. *Journal of Bone & Joint Surgery - American Volume.* 2014;96(18):e154.

3. Nelson CL, Jones RB, Wingert NC, Foltzer M, Bowen TR. Sonication of antibiotic spacers predicts failure during two-stage revision for prosthetic knee and hip infections.[Erratum appears in Clin Orthop Relat Res. 2014 Jul;472(7):2307]. *Clinical Orthopaedics & Related Research.* 2014;472(7):2208-2214.

4. Glynn A, Huang R, Mortazavi J, Parvizi J. The impact of patellar resurfacing in two-stage revision of the infected total knee arthroplasty. *Journal of Arthroplasty.* 2014;29(7):1439-1442.

5. Dietz MJ, Choi HR, Freiberg AA, Bedair H. Transfer of patient care during two-stage exchange for periprosthetic joint infection leads to inferior outcomes. *Journal of Arthroplasty.* 2014;29(7):1426-1429.

6. Sassoon AA, Nelms NJ, Trousdale RT. Intraoperative fracture during staged total knee reimplantation in the treatment of periprosthetic infection. *Journal of Arthroplasty.* 2014;29(7):1435-1438.

7. Le DH, Goodman SB, Maloney WJ, Huddleston JI. Current modes of failure in TKA: infection, instability, and stiffness predominate. *Clinical Orthopaedics & Related Research.* 2014;472(7):2197-2200.

8. Kapadia BH, McElroy MJ, Issa K, Johnson AJ, Bozic KJ, Mont MA. The economic impact of periprosthetic infections following total knee arthroplasty at a specialized tertiary-care center. *Journal of Arthroplasty.* 2014;29(5):929-932.

9. Aggarwal VK, Goyal N, Deirmengian G, Rangavajulla A, Parvizi J, Austin MS. Revision total knee arthroplasty in the young patient: is there trouble on the horizon? *Journal of Bone & Joint Surgery - American Volume.* 2014;96(7):536-542.

10. Meehan JP, Danielsen B, Kim SH, Jamali AA, White RH. Younger age is associated with a higher risk of early periprosthetic joint infection and aseptic mechanical failure after total knee arthroplasty. *Journal of Bone & Joint Surgery - American Volume.* 2014;96(7):529-535.

11. Guild GN, 3rd, Wu B, Scuderi GR. Articulating vs. Static antibiotic impregnated spacers in revision total knee arthroplasty for sepsis. A systematic review. *Journal of Arthroplasty.* 2014;29(3):558-563.

12. Pivec R, Naziri Q, Issa K, Banerjee S, Mont MA. Systematic review comparing static and articulating spacers used for revision of infected total knee arthroplasty. *Journal of Arthroplasty.* 2014;29(3):553-557.e551.

13. Scarponi S, Drago L, Romano D, et al. Cementless modular intramedullary nail without bone-on-bone fusion as a salvage procedure in chronically infected total knee prosthesis: long-term results. *International Orthopaedics.* 2014;38(2):413-418.

14. Edwards PK, Fehring TK, Hamilton WG, Perricelli B, Beaver WB, Odum SM. Are cementless stems more durable than cemented stems in two-stage revisions of infected total knee arthroplasties? *Clinical Orthopaedics & Related Research.* 2014;472(1):206-211.

15. Schairer WW, Vail TP, Bozic KJ. What are the rates and causes of hospital readmission after total knee arthroplasty? *Clinical Orthopaedics & Related Research.* 2014;472(1):181-187.

16. Baker P, Petheram TG, Kurtz S, Konttinen YT, Gregg P, Deehan D. Patient reported outcome measures after revision of the infected TKR: comparison of single versus two-stage revision. *Knee Surgery, Sports Traumatology, Arthroscopy.* 2013;21(12):2713-2720.

17. Cho WS, Byun SE, Cho WJ, Yoon YS, Dhurve K. Polymorphonuclear cell count on frozen section is not an absolute index of reimplantation in infected total knee arthroplasty. *Journal of Arthroplasty.* 2013;28(10):1874-1877.

18. Struelens B, Claes S, Bellemans J. Spacer-related problems in two-stage revision knee arthroplasty. *Acta Orthopaedica Belgica.* 2013;79(4):422-426.

19. Voleti PB, Baldwin KD, Lee G-C. Use of Static or Articulating Spacers for Infection Following Total Knee Arthroplasty A Systematic Literature Review. *Journal of Bone and Joint Surgery-American Volume.* 2013;95A(17):1594-1599.

20. Masters JP, Smith NA, Foguet P, Reed M, Parsons H, Sprowson AP. A systematic review of the evidence for single stage and two stage revision of infected knee replacement. *BMC Musculoskeletal Disorders.* 2013;14:222.

21. Carulli C, Villano M, Civinini R, Matassi F, Nistri L, Innocenti M. A novel technique to preserve range of motion in two-stage revision of infected total knee arthroplasty. *International Orthopaedics.* 2013;37(6):1069-1074.

22. Tay KS, Lo NN, Yeo SJ, Chia SL, Tay DK, Chin PL. Revision total knee arthroplasty: causes and outcomes. *Annals of the Academy of Medicine, Singapore.* 2013;42(4):178-183.

23. Nettrour JF, Polikandriotis JA, Bernasek TL, Gustke KA, Lyons ST. Articulating spacers for the treatment of infected total knee arthroplasty: effect of antibiotic combinations and concentrations. *Orthopedics.* 2013;36(1):e19-24.

24. Siddiqui MM, Lo NN, Ab Rahman S, Chin PL, Chia SL, Yeo SJ. Two-year outcome of early deep MRSA infections after primary total knee arthroplasty: a joint registry review. *Journal of Arthroplasty.* 2013;28(1):44-48.

25. Xiao-Gang Z, Shahzad K, Li C. One-stage total knee arthroplasty for patients with osteoarthritis of the knee and extra-articular deformity. *International Orthopaedics.* 2012;36(12):2457-2463.

26. Jaen F, Sanz-Gallardo MI, Arrazola MP, et al. [Multicentre study of infection incidence in knee prosthesis]. *Revista Espanola de Cirugia Ortopedica y Traumatologia.* 2012;56(1):38-45.

27. Romano CL, Gala L, Logoluso N, Romano D, Drago L. Two-stage revision of septic knee prosthesis with articulating knee spacers yields better infection eradication rate than one-stage or two-stage revision with static spacers. *Knee Surgery, Sports Traumatology, Arthroscopy.* 2012;20(12):2445-2453.

28. Munro JT, Garbuz DS, Masri BA, Duncan CP. Articulating antibiotic impregnated spacers in two-stage revision of infected total knee arthroplasty. *Journal of Bone & Joint Surgery - British Volume.* 2012;94(11 Suppl A):123-125.

29. Gulhane S, Vanhegan IS, Haddad FS. Single stage revision: regaining momentum. *Journal of Bone & Joint Surgery - British Volume.* 2012;94(11 Suppl A):120-122.

30. Cram P, Lu X, Kates SL, Singh JA, Li Y, Wolf BR. Total knee arthroplasty volume, utilization, and outcomes among Medicare beneficiaries, 1991-2010. *JAMA.* 2012;308(12):1227-1236.

31. Stroh DA, Johnson AJ, Naziri Q, Mont MA. Discrepancies between frozen and paraffin tissue sections have little effect on outcome of staged total knee arthroplasty revision for infection. *Journal of Bone & Joint Surgery - American Volume.* 2012;94(18):1662-1667.

32. Kim TW, Makani A, Choudhury R, Kamath AF, Lee GC. Patient-reported activity levels after successful treatment of infected total knee arthroplasty. *Journal of Arthroplasty.* 2012;27(8 Suppl):81-85.

33. Choi HR, Kwon YM, Burke DW, Rubash HE, Malchau H. The outcome of sequential repeated tibial tubercle osteotomy performed in 2-stage revision arthroplasty for infected total knee arthroplasty. *Journal of Arthroplasty.* 2012;27(8):1487-1491.

34. Choi HR, Burke D, Malchau H, Kwon YM. Utility of tibial tubercle osteotomy in the setting of periprosthetic infection after total knee arthroplasty. *International Orthopaedics.* 2012;36(8):1609-1613.

35. Jaekel DJ, Day JS, Klein GR, Levine H, Parvizi J, Kurtz SM. Do dynamic cement-on-cement knee spacers provide better function and activity during two-stage exchange? *Clinical Orthopaedics & Related Research.* 2012;470(9):2599-2604.

36. Rasouli MR, Harandi AA, Adeli B, Purtill JJ, Parvizi J. Revision total knee arthroplasty: infection should be ruled out in all cases. *Journal of Arthroplasty.* 2012;27(6):1239-1243.e1231-1232.

37. Malviya A, Bettinson K, Kurtz SM, Deehan DJ. When do patient-reported assessments peak after revision knee arthroplasty? *Clinical Orthopaedics & Related Research.* 2012;470(6):1728-1734.

38. Hwang BH, Yoon JY, Nam CH, et al. Fungal peri-prosthetic joint infection after primary total knee replacement. *Journal of Bone & Joint Surgery - British Volume.* 2012;94(5):656-659.

39. Singer J, Merz A, Frommelt L, Fink B. High rate of infection control with one-stage revision of septic knee prostheses excluding MRSA and MRSE. *Clinical Orthopaedics & Related Research.* 2012;470(5):1461-1471.

40. Sorli L, Puig L, Torres-Claramunt R, et al. The relationship between microbiology results in the second of a two-stage exchange procedure using cement spacers and the outcome after revision total joint replacement for infection: the use of sonication to aid bacteriological analysis. *Journal of Bone & Joint Surgery - British Volume.* 2012;94(2):249-253.

41. Nickinson RS, Board TN, Gambhir AK, Porter ML, Kay PR. Two stage revision knee arthroplasty for infection with massive bone loss. A technique to achieve spacer stability. *Knee.* 2012;19(1):24-27.

42. Whiteside LA, Nayfeh TA, LaZear R, Roy ME. Reinfected revised TKA resolves with an aggressive protocol and antibiotic infusion. *Clinical Orthopaedics & Related Research.* 2012;470(1):236-243.

43. Kalore NV, Maheshwari A, Sharma A, Cheng E, Gioe TJ. Is there a preferred articulating spacer technique for infected knee arthroplasty? A preliminary study. *Clinical Orthopaedics & Related Research.* 2012;470(1):228-235.

44. Macheras GA, Kateros K, Galanakos SP, Koutsostathis SD, Kontou E, Papadakis SA. The long-term results of a two-stage protocol for revision of an infected total knee replacement. *Journal of Bone & Joint Surgery - British Volume.* 2011;93(11):1487-1492.

45. Mortazavi SM, Vegari D, Ho A, Zmistowski B, Parvizi J. Two-stage exchange arthroplasty for infected total knee arthroplasty: predictors of failure. *Clinical Orthopaedics & Related Research.* 2011;469(11):3049-3054.

46. Macmull S, Bartlett W, Miles J, et al. Custom-made hinged spacers in revision knee surgery for patients with infection, bone loss and instability. *Knee.* 2010;17(6):403-406.

47. Yoo J, Lee S, Han C, Chang J. The modified static spacers using antibiotic-impregnated cement rod in two-stage revision for infected total knee arthroplasty. *Clinics in Orthopedic Surgery.* 2011;3(3):245-248.

48. Mortazavi SM, Molligan J, Austin MS, Purtill JJ, Hozack WJ, Parvizi J. Failure following revision total knee arthroplasty: infection is the major cause. *International Orthopaedics.* 2011;35(8):1157-1164.

49. Senneville E, Joulie D, Legout L, et al. Outcome and predictors of treatment failure in total hip/knee prosthetic joint infections due to Staphylococcus aureus. *Clinical Infectious Diseases.* 2011;53(4):334-340.

50. Gooding CR, Masri BA, Duncan CP, Greidanus NV, Garbuz DS. Durable infection control and function with the PROSTALAC spacer in two-stage revision for infected knee arthroplasty. *Clinical Orthopaedics & Related Research.* 2011;469(4):985-993.

51. Gardner J, Gioe TJ, Tatman P. Can this prosthesis be saved?: implant salvage attempts in infected primary TKA. *Clinical Orthopaedics & Related Research.* 2011;469(4):970-976.

52. Zywiel MG, Johnson AJ, Stroh DA, Martin J, Marker DR, Mont MA. Prophylactic oral antibiotics reduce reinfection rates following two-stage revision total knee arthroplasty. *International Orthopaedics.* 2011;35(1):37-42.

53. Sherrell JC, Fehring TK, Odum S, et al. The Chitranjan Ranawat Award: fate of two-stage reimplantation after failed irrigation and debridement for periprosthetic knee infection. *Clinical Orthopaedics & Related Research.* 2011;469(1):18-25.

54. Qiu XS, Sun X, Chen DY, Xu ZH, Jiang Q. Application of an articulating spacer in two-stage revision for severe infection after total knee arthroplasty. *Orthopaedic Audio-Synopsis Continuing Medical Education [Sound Recording].* 2010;2(4):299-304.

55. Romano CL, Romano D, Logoluso N, Meani E. Septic versus aseptic hip revision: how different? *Journal of Orthopaedics & Traumatology.* 2010;11(3):167-174.

56. Lee J, Kang CI, Lee JH, et al. Risk factors for treatment failure in patients with prosthetic joint infections.[Erratum appears in J Hosp Infect. 2010 Nov;76(3):281]. *Journal of Hospital Infection.* 2010;75(4):273-276.

57. Estes CS, Beauchamp CP, Clarke HD, Spangehl MJ. A two-stage retention debridement protocol for acute periprosthetic joint infections. *Clinical Orthopaedics & Related Research.* 2010;468(8):2029-2038.

58. Hwang SC, Kong JY, Nam DC, et al. Revision total knee arthroplasty with a cemented posterior stabilized, condylar constrained or fully constrained prosthesis: a minimum 2-year follow-up analysis. *Clinics in Orthopedic Surgery.* 2010;2(2):112-120.

59. Hossain F, Patel S, Haddad FS. Midterm assessment of causes and results of revision total knee arthroplasty. *Clinical Orthopaedics & Related Research.* 2010;468(5):1221-1228.

60. Kosters K, van Crevel R, Sturm PD, et al. Treatment of knee prosthesis infections: evaluation of 15 patients over a 5-year period. *International Orthopaedics.* 2009;33(5):1249-1254.

61. Morgan PM, Sharkey P, Ghanem E, et al. The value of intraoperative Gram stain in revision total knee arthroplasty.[Erratum appears in J Bone Joint Surg Am. 2010 Feb;92(2):442]. *Journal of Bone & Joint Surgery - American Volume.* 2009;91(9):2124-2129.

62. Azzam K, McHale K, Austin M, Purtill JJ, Parvizi J. Outcome of a second two-stage reimplantation for periprosthetic knee infection. *Clinical Orthopaedics & Related Research.* 2009;467(7):1706-1714.

63. Savarino L, Tigani D, Baldini N, Bochicchio V, Giunti A. Pre-operative diagnosis of infection in total knee arthroplasty: an algorithm. *Knee Surgery, Sports Traumatology, Arthroscopy.* 2009;17(6):667-675.

64. Chiu FY, Lin CF. Antibiotic-impregnated cement in revision total knee arthroplasty. A prospective cohort study of one hundred and eighty-three knees. *Journal of Bone & Joint Surgery - American Volume.* 2009;91(3):628-633.

65. Bauman RD, Lewallen DG, Hanssen AD. Limitations of structural allograft in revision total knee arthroplasty. *Clinical Orthopaedics & Related Research.* 2009;467(3):818-824.

66. Luscombe JC, Theivendran K, Abudu A, Carter SR. The relative safety of one-stage bilateral total knee arthroplasty. *International Orthopaedics.* 2009;33(1):101-104.

67. Mutimer J, Gillespie G, Lovering AM, Porteous AJ. Measurements of in vivo intra-articular gentamicin levels from antibiotic loaded articulating spacers in revision total knee replacement. *Knee.* 2009;16(1):39-41.

68. Pun SY, Ries MD. Effect of gender and preoperative diagnosis on results of revision total knee arthroplasty. *Clinical Orthopaedics & Related Research.* 2008;466(11):2701-2705.

69. Betsch BY, Eggli S, Siebenrock KA, Taeuber MG, Muehlemann K. Treatment of joint prosthesis infection in accordance with current recommendations improves outcome. *Clinical Infectious Diseases.* 2008;46(8):1221-1226.

70. Barrack RL, Aggarwal A, Burnett RS, et al. The fate of the unexpected positive intraoperative cultures after revision total knee arthroplasty. *Journal of Arthroplasty.* 2007;22(6 Suppl 2):94-99.

71. Ghanem E, Restrepo C, Joshi A, Hozack W, Sharkey P, Parvizi J. Periprosthetic infection does not preclude good outcome for revision arthroplasty. *Clinical Orthopaedics & Related Research.* 2007;461:54-59.

72. Mittal Y, Fehring TK, Hanssen A, Marculescu C, Odum SM, Osmon D. Two-stage reimplantation for periprosthetic knee infection involving resistant organisms. *Journal of Bone & Joint Surgery - American Volume.* 2007;89(6):1227-1231.

73. Whiteside LA. Cementless fixation in revision total knee arthroplasty. *Clinical Orthopaedics & Related Research.* 2006;446:140-148.

74. Bare J, MacDonald SJ, Bourne RB. Preoperative evaluations in revision total knee arthroplasty. *Clinical Orthopaedics & Related Research.* 2006;446:40-44.

75. Pietsch M, Hofmann S, Wenisch C. Treatment of deep infection of total knee arthroplasty using a two-stage procedure. *Operative Orthopadie und Traumatologie.* 2006;18(1):66-87.

76. MacAvoy MC, Ries MD. The ball and socket articulating spacer for infected total knee arthroplasty. *Journal of Arthroplasty.* 2005;20(6):757-762.

77. Peters CL, Erickson J, Kloepper RG, Mohr RA. Revision total knee arthroplasty with modular components inserted with metaphyseal cement and stems without cement. *Journal of Arthroplasty.* 2005;20(3):302-308.

78. Pradhan NR, Bale L, Kay P, Porter ML. Salvage revision total knee replacement using the Endo-Model rotating hinge prosthesis. *Knee.* 2004;11(6):469-473.

79. Springer BD, Lee GC, Osmon D, Haidukewych GJ, Hanssen AD, Jacofsky DJ. Systemic safety of high-dose antibiotic-loaded cement spacers after resection of an infected total knee arthroplasty. *Clinical Orthopaedics & Related Research.* 2004(427):47-51.

80. Hendel D, Weisbort M, Garti A. "Wandering resident" surgical exposure for 1- or 2-stage revision arthroplasty in stiff aseptic and septic knee arthroplasty. *Journal of Arthroplasty.* 2004;19(6):757-759.

81. Blom AW, Brown J, Taylor AH, Pattison G, Whitehouse S, Bannister GC. Infection after total knee arthroplasty. *Journal of Bone & Joint Surgery - British Volume.* 2004;86(5):688-691.

82. Buechel FF. The infected total knee arthroplasty: just when you thought it was over. *Journal of Arthroplasty.* 2004;19(4 Suppl 1):51-55.

83. Pitto RP, Spika IA. Antibiotic-loaded bone cement spacers in two-stage management of infected total knee arthroplasty. *International Orthopaedics.* 2004;28(3):129-133.

84. Wang CJ, Hsieh MC, Huang TW, Wang JW, Chen HS, Liu CY. Clinical outcome and patient satisfaction in aseptic and septic revision total knee arthroplasty. *Knee.* 2004;11(1):45-49.

85. Meek RM, Masri BA, Dunlop D, et al. Patient satisfaction and functional status after treatment of infection at the site of a total knee arthroplasty with use of the PROSTALAC articulating spacer. *Journal of Bone & Joint Surgery - American Volume.* 2003;85-A(10):1888-1892.

86. Shannon BD, Klassen JF, Rand JA, Berry DJ, Trousdale RT. Revision total knee arthroplasty with cemented components and uncemented intramedullary stems. *Journal of Arthroplasty.* 2003;18(7 Suppl 1):27-32.

87. Nazarian DG, de Jesus D, McGuigan F, Booth RE, Jr. A two-stage approach to primary knee arthroplasty in the infected arthritic knee. *Journal of Arthroplasty.* 2003;18(7 Suppl 1):16-21.

88. Luria S, Kandel L, Segal D, Liebergall M, Mattan Y. Revision total knee arthroplasty. *Israel Medical Association Journal: Imaj.* 2003;5(8):552-555.

89. Silva M, Tharani R, Schmalzried TP. Results of direct exchange or debridement of the infected total knee arthroplasty. *Clinical Orthopaedics & Related Research.* 2002(404):125-131.

90. Wang CJ, Huang TW, Wang JW, Chen HS. The often poor clinical outcome of infected total knee arthroplasty. *Journal of Arthroplasty.* 2002;17(5):608-614.

91. Insall JN, Thompson FM, Brause BD. Two-stage reimplantation for the salvage of infected total knee arthroplasty. 1983. *Journal of Bone & Joint Surgery - American Volume.* 2002;84-A(3):490.

92. Bohm I, Landsiedl F. Revision surgery after failed unicompartmental knee arthroplasty: a study of 35 cases. *Journal of Arthroplasty.* 2000;15(8):982-989.

93. Pagnano M, Cushner FD, Hansen A, Scuderi GR, Scott WN. Blood management in two-stage revision knee arthroplasty for deep prosthetic infection. *Clinical Orthopaedics & Related Research.* 1999(367):238-242.

94. Mow CS, Wiedel JD. Revision total knee arthroplasty using the porous-coated anatomic revision prosthesis: six- to twelve-year results.[Erratum appears in J Arthroplasty 1999 Feb;14(2):264]. *Journal of Arthroplasty.* 1998;13(6):681-686.

95. Backe HA, Jr., Wolff DA, Windsor RE. Total knee replacement infection after 2-stage reimplantation: results of subsequent 2-stage reimplantation. *Clinical Orthopaedics & Related Research.* 1996(331):125-131.

96. Chimento GF, Finger S, Barrack RL. Gram stain detection of infection during revision arthroplasty. *Journal of Bone & Joint Surgery - British Volume.* 1996;78(5):838-839.

97. Hanssen AD, Trousdale RT, Osmon DR. Patient outcome with reinfection following reimplantation for the infected total knee arthroplasty. *Clinical Orthopaedics & Related Research.* 1995(321):55-67.

98. Vince KG, Long W. Revision knee arthroplasty. The limits of press fit medullary fixation. *Clinical Orthopaedics & Related Research.* 1995(317):172-177.

99. Takahashi Y, Gustilo RB. Nonconstrained implants in revision total knee arthroplasty. *Clinical Orthopaedics & Related Research.* 1994(309):156-162.

100. Bengtson S, Knutson K, Lidgren L. TREATMENT OF INFECTED KNEE ARTHROPLASTY. *Clinical Orthopaedics and Related Research.* 1989(245):173-178.

101. Wilde AH, Ruth JT. Two-stage reimplantation in infected total knee arthroplasty. *Clinical Orthopaedics & Related Research.* 1988(236):23-35.

102. Bengtson S, Knutson K, Lidgren L. Revision of infected knee arthroplasty. *Acta Orthopaedica Scandinavica.* 1986;57(6):489-494.

103. Elia EA, Lotke PA. Results of revision total knee arthroplasty associated with significant bone loss. *Clinical Orthopaedics and Related Research.* 1991(271):114-121.

104. Stambough JB, Clohisy JC, Barrack RL, Nunley RM, Keeney JA. Increased risk of failure following revision total knee replacement in patients aged 55 years and younger. *Bone & Joint Journal.* 2014;96B(12):1657-1662.

105. Oheim R, Gille J, Schoop R, et al. Surgical therapy of extensive knee joint empyema: mid-term results after two-stage versus one-stage procedures. *Knee Surgery Sports Traumatology Arthroscopy.* 2014;22(12):3150-3156.

106. Dieterich JD, Fields AC, Moucha CS. Short Term Outcomes of Revision Total Knee Arthroplasty. *Journal of Arthroplasty.* 2014;29(11):2163-2166.

107. Webb JE, Schleck CD, Larson DR, Lewallen DG, Trousdale RT. Mortality of Elderly Patients After Two-Stage Reimplantation for Total Joint Infection: A Case-Control Study. *Journal of Arthroplasty.* 2014;29(11):2206-2210.

108. Choi HR, Bedair H. Mortality following revision total knee arthroplasty: a matched cohort study of septic versus aseptic revisions. *Journal of Arthroplasty.* 2014;29(6):1216-1218.

109. Olivecrona C, Lapidus LJ, Benson L, Blomfeldt R. Tourniquet time affects postoperative complications after knee arthroplasty. *International Orthopaedics.* 2013;37(5):827-832.

110. Brunnekreef J, Hannink G, Malefijt Mde W. Recovery of knee mobility after a static or mobile spacer in total knee infection. *Acta Orthopaedica Belgica.* 2013;79(1):83-89.

111. Kim YH, Choi Y, Kim JS. Treatment based on the type of infected TKA improves infection control. *Clinical Orthopaedics & Related Research.* 2011;469(4):977-984.

112. Nickinson RS, Board TN, Gambhir AK, Porter ML, Kay PR. The microbiology of the infected knee arthroplasty. *International Orthopaedics.* 2010;34(4):505-510.

113. Bejon P, Berendt A, Atkins BL, et al. Two-stage revision for prosthetic joint infection: predictors of outcome and the role of reimplantation microbiology. *Journal of Antimicrobial Chemotherapy.* 2010;65(3):569-575.

114. Volin SJ, Hinrichs SH, Garvin KL. Two-stage reimplantation of total joint infections: a comparison of resistant and non-resistant organisms. *Clinical Orthopaedics & Related Research.* 2004(427):94-100.

115. Cushner FD, Locker JR, Hanssen AD, et al. Use of recombinant human erythropoietin in two-stage total knee arthroplasty for infection. *Clinical Orthopaedics & Related Research.* 2001(392):116-123.

116. Atkins BL, Athanasou N, Deeks JJ, et al. Prospective evaluation of criteria for microbiological diagnosis of prosthetic-joint infection at revision arthroplasty. The OSIRIS Collaborative Study Group. *Journal of Clinical Microbiology.* 1998;36(10):2932-2939.

117. Goksan SB, Freeman MA. One-stage reimplantation for infected total knee arthroplasty. *Journal of Bone & Joint Surgery - British Volume.* 1992;74(1):78-82.

118. Smith CS, Zitzke RC, Duffy GP. A new technique for articulated cement spacers in infected total knee arthroplasty. *American Journal of Orthopedics (Chatham, Nj).* 2012;41(12):E163-165.

119. Radoicic D, Popovic Z, Barjaktarovic R, Marinkovic J. Infected total knee arthroplasty treatment outcome analysis. *Vojnosanitetski Pregled.* 2012;69(6):504-509.

120. Kou B, Lu H, Yuan Y, Yan T, Zhou D. [Clinical analysis of 13 infected total knee replacements]. *Chung-Hua Wai Ko Tsa Chih [Chinese Journal of Surgery].* 2000;38(4):253-255.

121. Haas SB, Insall JN, Montgomery W, 3rd, Windsor RE. Revision total knee arthroplasty with use of modular components with stems inserted without cement. *Journal of Bone & Joint Surgery - American Volume.* 1995;77(11):1700-1707.

122. Drobny TK, Munzinger UK, Chomiak J. [2-stage exchange in the treatment of infected knee prosthesis]. *Orthopade.* 1995;24(4):360-366.

123. Kramhoft M, Bodtker S, Carlsen A. Outcome of infected total knee arthroplasty. *Journal of Arthroplasty.* 1994;9(6):617-621.

124. Drobny TK, Munzinger U. [Problems of infected knee prosthesis]. *Orthopade.* 1991;20(3):239-243.

125. Insall JN, Thompson FM, Brause BD. Two-stage reimplantation for the salvage of infected total knee arthroplasty. *Journal of Bone & Joint Surgery - American Volume.* 1983;65(8):1087-1098.

126. Rand JA, Bryan RS. Reimplantation for the salvage of an infected total knee arthroplasty. *Journal of Bone & Joint Surgery - American Volume.* 1983;65(8):1081-1086.

127. Kendoff D, Gehrke T. Surgical Management of Periprosthetic Joint Infection: One-Stage Exchange. *Journal of Knee Surgery.* 2014;27(4):273-278.

128. Jhao C, Jiang CC. Two-stage reimplantation without cement spacer for septic total knee replacement. *Journal of the Formosan Medical Association.* 2003;102(1):37-41.

129. Booth Jr RE, Lotke PA. The results of spacer block technique in revision of infected total knee arthroplasty. *Clinical Orthopaedics and Related Research.* 1989(248):57-60.

130. Gusso MI, Capone A, Civinini R, Scoccianti G. The spacer block technique in revision of total knee arthroplasty with septic loosening. *Chirurgia Degli Organi di Movimento.* 1995;80(1):21-27.

131. Lu H, Kou B, Lin J. [One-stage reimplantation for the salvage of total knee arthroplasty complicated by infection]. *Chung-Hua Wai Ko Tsa Chih [Chinese Journal of Surgery].* 1997;35(8):456-458.

132. Kirschner S, Werner A, Walther M, Rader CP, Gohlke FE. Versorgung mit anatomischen Spacern bei chronischem endoprotheseinfekt am Knie. *Orthop Praxis.* 2000;36(7):422-426.

133. Freeman MA, Sudlow RA, Casewell MW, Radcliff SS. The management of infected total knee replacements. *Journal of Bone & Joint Surgery - British Volume.* 1985;67(5):764-768.

134. Kirpalani PA, In Y, Choi NY, Koh HS, Kim JM, Han CW. Two-stage total knee arthroplasty for non-salvageable septic arthritis in diabetes mellitus patients. *Acta Orthopaedica Belgica.* 2005;71(3):315-320.

135. Goldstein WM, Kopplin M, Wall R, Berland K. Temporary articulating methylmethacrylate antibiotic spacer (TAMMAS). A new method of intraoperative manufacturing of a custom articulating spacer. *Journal of Bone & Joint Surgery - American Volume.* 2001;83-A Suppl 2 Pt 2:92-97.

**Appendix E.** Reference list of included studies

1. Bengtson S, Knutson K. The infected knee arthroplasty. A 6-year follow-up of 357 cases. *Acta orthopaedica Scandinavica.* 1991;62(4):301-311.

2. von Foerster G, Kluber D, Kabler U. Mid- to long-term results after treatment of 118 cases of periprosthetic infections after knee joint replacement using one-stage exchange surgery. *Orthopade.* 1991;20(3):244-252.

3. Scott IR, Stockley I, Getty CJ. Exchange arthroplasty for infected knee replacements. A new two-stage method. *Journal of Bone & Joint Surgery - British Volume.* 1993;75(1):28-31.

4. Buechel FF, Femino FP, D'Alessio J. Primary exchange revision arthroplasty for infected total knee replacement: a long-term study. *Am J Orthop.* 2004;33(4):190-198; discussion 198.

5. Sofer D, Regenbrecht B, Pfeil J. Early results of one-stage septic revision arthroplasties with antibiotic-laden cement. A clinical and statistical analysis. *Orthopade.* 2005;34(6):592-602.

6. Bauer T, Piriou P, Lhotellier L, Leclerc P, Mamoudy P, Lortat-Jacob A. [Results of reimplantation for infected total knee arthroplasty: 107 cases]. *Revue de Chirurgie Orthopedique et Reparatrice de l Appareil Moteur.* 2006;92(7):692-700.

7. Jenny JY, Barbe B, Gaudias J, Boeri C, Argenson JN. High infection control rate and function after routine one-stage exchange for chronically infected TKA. *Clinical Orthopaedics & Related Research.* 2013;471(1):238-243.

8. van Kempen RW, Schimmel JJ, van Hellemondt GG, Vandenneucker H, Wymenga AB. Reason for revision TKA predicts clinical outcome: prospective evaluation of 150 consecutive patients with 2-years followup. *Clin Orthop.* 2013;471(7):2296-2302.

9. Tibrewal S, Malagelada F, Jeyaseelan L, Posch F, Scott G. Single-stage revision for the infected total knee replacement: results from a single centre. *Bone Joint J.* 2014;96-B(6):759-764.

10. Haddad FS, Sukeik M, Alazzawi S. Is single-stage revision according to a strict protocol effective in treatment of chronic knee arthroplasty infections? *Clin Orthop.* 2015;473(1):8-14.

11. Walker RH, Schurman DJ. Management of infected total knee arthroplasties. *Clin Orthop.* 1984(186):81-89.

12. Borden LS, Gearen PF. Infected total knee arthroplasty. A protocol for management. *Journal of Arthroplasty.* 1987;2(1):27-36.

13. Rosenberg AG, Haas B, Barden R, Marquez D, Landon GC, Galante JO. Salvage of infected total knee arthroplasty. *Clin Orthop.* 1988(226):29-33.

14. Morrey BF, Westholm F, Schoifet S, Rand JA, Bryan RS. Long-term results of various treatment options for infected total knee arthroplasty. *Clin Orthop.* 1989(248):120-128.

15. Wilson MG, Kelley K, Thornhill TS. Infection as a complication of total knee-replacement arthroplasty. Risk factors and treatment in sixty-seven cases. *J Bone Joint Surg Am.* 1990;72(6):878-883.

16. Windsor RE, Insall JN, Urs WK, Miller DV, Brause BD. Two-stage reimplantation for the salvage of total knee arthroplasty complicated by infection. Further follow-up and refinement of indications. *Journal of Bone & Joint Surgery - American Volume.* 1990;72(2):272-278.

17. Henderson MH, Jr., Booth RE, Jr. The use of an antibiotic-impregnated spacer block for revision of the septic total knee arthroplasty. *Seminars in Arthroplasty.* 1991;2(1):34-39.

18. Whiteside LA. Treatment of infected total knee arthroplasty. *Clin Orthop.* 1994(299):169-172.

19. Hanssen AD, Rand JA, Osmon DR. Treatment of the infected total knee arthroplasty with insertion of another prosthesis. The effect of antibiotic-impregnated bone cement. *Clin Orthop.* 1994(309):44-55.

20. Masri BA, Kendall RW, Duncan CP, Beauchamp CP, McGraw RW, Bora B. Two-stage exchange arthroplasty using a functional antibiotic-loaded spacer in the treatment of the infected knee replacement: the Vancouver experience. *Seminars in Arthroplasty.* 1994;5(3):122-136.

21. Bose WJ, Gearen PF, Randall JC, Petty W. Long-term outcome of 42 knees with chronic infection after total knee arthroplasty. *Clinical Orthopaedics & Related Research.* 1995(319):285-296.

22. Hofmann AA, Kane KR, Tkach TK, Plaster RL, Camargo MP. Treatment of infected total knee arthroplasty using an articulating spacer. *Clin Orthop.* 1995(321):45-54.

23. Goldman RT, Scuderi GR, Insall JN. 2-stage reimplantation for infected total knee replacement. *Clinical Orthopaedics & Related Research.* 1996(331):118-124.

24. Calton TF, Fehring TK, Griffin WL. Bone loss associated with the use of spacer blocks in infected total knee arthroplasty. *Clinical Orthopaedics & Related Research.* 1997(345):148-154.

25. Gacon G, Laurencon M, Van de Velde D, Giudicelli DP. [Two stages reimplantation for infection after knee arthroplasty. Apropos of a series of 29 cases]. *Revue de Chirurgie Orthopedique et Reparatrice de l Appareil Moteur.* 1997;83(4):313-323.

26. McPherson EJ, Patzakis MJ, Gross JE, Holtom PD, Song M, Dorr LD. Infected total knee arthroplasty. Two-stage reimplantation with a gastrocnemius rotational flap. *Clinical Orthopaedics & Related Research.* 1997(341):73-81.

27. Hirakawa K, Stulberg BN, Wilde AH, Bauer TW, Secic M. Results of 2-stage reimplantation for infected total knee arthroplasty. *Journal of Arthroplasty.* 1998;13(1):22-28.

28. Jerosch J, Mersmann M, Fuchs S. [Treatment modalities in infected knee alloarthroplasties]. *Zeitschrift fur Orthopadie und Ihre Grenzgebiete.* 1999;137(1):61-66.

29. Lecuire F, Rubini J, Basso M, Benareau I. Traction-mobilization in 2-stage treatment of infected total knee prosthesis. Apropos of 12 cases. *Revue de Chirurgie Orthopedique et Reparatrice de l Appareil Moteur.* 1999;85(6):640-645.

30. Segawa H, Tsukayama DT, Kyle RF, Becker DA, Gustilo RB. Infection after total knee arthroplasty. A retrospective study of the treatment of eighty-one infections. *J Bone Joint Surg Am.* 1999;81(10):1434-1445.

31. Barrack RL, Engh G, Rorabeck C, Sawhney J, Woolfrey M. Patient satisfaction and outcome after septic versus aseptic revision total knee arthroplasty. *Journal of Arthroplasty.* 2000;15(8):990-993.

32. Fehring TK, Odum S, Calton TF, Mason JB. Articulating versus static spacers in revision total knee arthroplasty for sepsis. The Ranawat Award. *Clinical Orthopaedics & Related Research.* 2000(380):9-16.

33. Haddad FS, Masri BA, Campbell D, McGraw RW, Beauchamp CP, Duncan CP. The PROSTALAC functional spacer in two-stage revision for infected knee replacements. Prosthesis of antibiotic-loaded acrylic cement. *Journal of Bone & Joint Surgery - British Volume.* 2000;82(6):807-812.

34. Mont MA, Waldman BJ, Hungerford DS. Evaluation of preoperative cultures before second-stage reimplantation of a total knee prosthesis complicated by infection. A comparison-group study. *Journal of Bone & Joint Surgery - American Volume.* 2000;82-A(11):1552-1557.

35. Lonner JH, Beck TD, Jr., Rees H, Roullet M, Lotke PA. Results of two-stage revision of the infected total knee arthroplasty. *American Journal of Knee Surgery.* 2001;14(1):65-67.

36. Emerson RH, Jr., Muncie M, Tarbox TR, Higgins LL. Comparison of a static with a mobile spacer in total knee infection. *Clin Orthop.* 2002(404):132-138.

37. Husted H, Toftgaard Jensen T. Clinical outcome after treatment of infected primary total knee arthroplasty. *Acta Orthop Belg.* 2002;68(5):500-507.

38. Siebel T, Kelm J, Porsch M, Regitz T, Neumann WH. Two-stage exchange of infected knee arthroplasty with an prosthesis-like interim cement spacer. *Acta Orthop Belg.* 2002;68(2):150-156.

39. Pietsch M, Wenisch C, Traussnig S, Trnoska R, Hofmann S. [Temporary articulating spacer with antibiotic-impregnated cement for an infected knee endoprosthesis]. *Orthopade.* 2003;32(6):490-497.

40. Evans RP. Successful treatment of total hip and knee infection with articulating antibiotic components: a modified treatment method. *Clin Orthop.* 2004;427:37-46.

41. Haleem AA, Berry DJ, Hanssen AD. Mid-term to long-term followup of two-stage reimplantation for infected total knee arthroplasty. *Clinical Orthopaedics & Related Research.* 2004(428):35-39.

42. Meek RMD, Dunlop D, Garbuz DS, McGraw R, Greidanus NV, Masri BA. Patient satisfaction and functional status after aseptic versus septic revision total knee arthroplasty using the PROSTALAC articulating spacer. *Journal of Arthroplasty.* 2004;19(7):874-879.

43. Durbhakula SM, Czajka J, Fuchs MD, Uhl RL. Antibiotic-loaded articulating cement spacer in the 2-stage exchange of infected total knee arthroplasty. *Journal of Arthroplasty.* 2004;19(6):768-774.

44. Cuckler JM. The infected total knee: management options. *Journal of Arthroplasty.* 2005;20(4 Suppl 2):33-36.

45. Pitto RP, Castelli CC, Ferrari R, Munro J. Pre-formed articulating knee spacer in two-stage revision for the infected total knee arthroplasty. *International Orthopaedics.* 2005;29(5):305-308.

46. Musil D, Stehlik J, Starek M. [Our experience with revision total knee arthroplasty]. *Acta Chirurgiae Orthopaedicae et Traumatologiae Cechoslovaca.* 2005;72(1):6-15.

47. Hoad-Reddick DA, Evans CR, Norman P, Stockley I. Is there a role for extended antibiotic therapy in a two-stage revision of the infected knee arthroplasty? *Journal of Bone & Joint Surgery - British Volume.* 2005;87(2):171-174.

48. Hofmann AA, Goldberg T, Tanner AM, Kurtin SM. Treatment of infected total knee arthroplasty using an articulating spacer: 2- to 12-year experience. *Clin Orthop.* 2005(430):125-131.

49. Huang HT, Su JY, Chen SK. The results of articulating spacer technique for infected total knee arthroplasty. *J Arthroplasty.* 2006;21(8):1163-1168.

50. Hart WJ, Jones RS. Two-stage revision of infected total knee replacements using articulating cement spacers and short-term antibiotic therapy. *Journal of Bone & Joint Surgery - British Volume.* 2006;88(8):1011-1015.

51. Jamsen E, Sheng P, Halonen P, et al. Spacer prostheses in two-stage revision of infected knee arthroplasty. *International Orthopaedics.* 2006;30(4):257-261.

52. Souillac V, Costes S, Aunoble S, Langlois V, Dutronc H, Chauveaux D. [Evaluation of an articulated spacer for two-stage reimplantation for infected total knee arthroplasty: 28 cases]. *Revue de Chirurgie Orthopedique et Reparatrice de l Appareil Moteur.* 2006;92(5):485-489.

53. Trezies A, Parish E, Dixon P, Cross M. The use of an articulating spacer in the management of infected total knee arthroplasties. *J Arthroplasty.* 2006;21(5):702-704.

54. Laffer RR, Graber P, Ochsner PE, Zimmerli W. Outcome of prosthetic knee-associated infection: evaluation of 40 consecutive episodes at a single centre. *Clin Microbiol Infect.* 2006;12(5):433-439.

55. Cordero-Ampuero J, Esteban J, Garcia-Cimbrelo E, Munuera L, Escobar R. Low relapse with oral antibiotics and two-stage exchange for late arthroplasty infections in 40 patients after 2-9 years. *Acta Orthop.* 2007;78(4):511-519.

56. Freeman MG, Fehring TK, Odum SM, Fehring K, Griffin WL, Mason JB. Functional Advantage of Articulating Versus Static Spacers in 2-Stage Revision for Total Knee Arthroplasty Infection. *The Journal of Arthroplasty.* 2007;22(8):1116-1121.

57. Hsu YC, Cheng HC, Ng TP, Chiu KY. Antibiotic-loaded cement articulating spacer for 2-stage reimplantation in infected total knee arthroplasty: a simple and economic method. *Journal of Arthroplasty.* 2007;22(7):1060-1066.

58. Pascale V, Pascale W. Custom-made articulating spacer in two-stage revision total knee arthroplasty. An early follow-up of 14 cases of at least 1 year after surgery. *HSS journal : the musculoskeletal journal of Hospital for Special Surgery.* 2007;3(2):159-163.

59. Thabe H, Schill S. Two-stage reimplantation with an application spacer and combined with delivery of antibiotics in the management of prosthetic joint infection. *Oper.* 2007;19(1):78-100.

60. Babis GC, Zahos KA, Tsailas P, Karaliotas GI, Kanellakopoulou K, Soucacos PN. Treatment of stage III-A-1 and III-B-1 periprosthetic knee infection with two-stage exchange arthroplasty and articulating spacer. *Journal of Surgical Orthopaedic Advances.* 2008;17(3):173-178.

61. Hsu C-S, Hsu C-C, Wang J-W, Lin P-C. Two-stage revision of infected total knee arthroplasty using an antibiotic-impregnated static cement-spacer. *Chang Gung Medical Journal.* 2008;31(6):583-591.

62. Villanueva-Martinez M, Rios-Luna A, Pereiro J, Fahandez-Saddi H, Villamor A. Hand-made articulating spacers in two-stage revision for infected total knee arthroplasty: good outcome in 30 patients. *Acta Orthopaedica.* 2008;79(5):674-682.

63. Anderson JA, Sculco PK, Heitkemper S, Mayman DJ, Bostrom MP, Sculco TP. An articulating spacer to treat and mobilize patients with infected total knee arthroplasty. *Journal of Arthroplasty.* 2009;24(4):631-635.

64. Ghanem E, Azzam K, Seeley M, Joshi A, Parvizi J. Staged revision for knee arthroplasty infection: what is the role of serologic tests before reimplantation? *Clinical Orthopaedics & Related Research.* 2009;467(7):1699-1705.

65. Peters CL, Erickson JA, Gililland JM. Clinical and radiographic results of 184 consecutive revision total knee arthroplasties placed with modular cementless stems. *J Arthroplasty.* 2009;24(6 Suppl):48-53.

66. Pietsch M, Wenisch C, Hofmann S. [Treatment of infected total knee arthroplasty. 2-5-year results following two-stage reimplantation]. *Orthopade.* 2009;38(4):348-354.

67. Su Y-P, Lee OK, Chen W-M, Chen T-H. A facile technique to make articulating spacers for infected total knee arthroplasty. *Journal of the Chinese Medical Association: JCMA.* 2009;72(3):138-145.

68. Cordero-Ampuero J, Esteban J, Garcia-Rey E. Results after late polymicrobial, gram-negative, and methicillin-resistant infections in knee arthroplasty. *Clin Orthop.* 2010;468(5):1229-1236.

69. Ocguder A, Firat A, Tecimel O, Solak S, Bozkurt M. Two-stage total infected knee arthroplasty treatment with articulating cement spacer. *Arch Orthop Trauma Surg.* 2010;130(6):719-725.

70. Patil N, Lee K, Huddleston JI, Harris AH, Goodman SB. Aseptic versus septic revision total knee arthroplasty: patient satisfaction, outcome and quality of life improvement. *Knee.* 2010;17(3):200-203.

71. Kurd MF, Ghanem E, Steinbrecher J, Parvizi J. Two-stage exchange knee arthroplasty: does resistance of the infecting organism influence the outcome? *Clin Orthop.* 2010;468(8):2060-2066.

72. Mortazavi SM, Schwartzenberger J, Austin MS, Purtill JJ, Parvizi J. Revision total knee arthroplasty infection: incidence and predictors. *Clinical Orthopaedics & Related Research.* 2010;468(8):2052-2059.

73. Shen H, Zhang X, Jiang Y, et al. Intraoperatively-made cement-on-cement antibiotic-loaded articulating spacer for infected total knee arthroplasty. *Knee.* 2010;17(6):407-411.

74. Park SJ, Song EK, Seon JK, Yoon TR, Park GH. Comparison of static and mobile antibiotic-impregnated cement spacers for the treatment of infected total knee arthroplasty. *Int Orthop.* 2010;34(8):1181-1186.

75. Ritter MA, Farris A. Outcome of infected total joint replacement. *Orthopedics.* 2010;33(3).

76. Westrich GH, Walcott-Sapp S, Bornstein LJ, Bostrom MP, Windsor RE, Brause BD. Modern treatment of infected total knee arthroplasty with a 2-stage reimplantation protocol. *J Arthroplasty.* 2010;25(7):1015-1021, 1021 e1011-1012.

77. Cabo J, Euba G, Saborido A, et al. Clinical outcome and microbiological findings using antibiotic-loaded spacers in two-stage revision of prosthetic joint infections. *J Infect.* 2011;63(1):23-31.

78. Choi HR, von Knoch F, Zurakowski D, Nelson SB, Malchau H. Can implant retention be recommended for treatment of infected TKA? *Clin Orthop.* 2011;469(4):961-969.

79. Ferrari R, Castelli CC, Gregis G. Preformed articulated knee spacer for the infected TKA: more than 10 years experience. *European Cells and Materials* 2011;21:47.

80. Garg P, Ranjan R, Bandyopadhyay U, Chouksey S, Mitra S, Gupta SK. Antibiotic-impregnated articulating cement spacer for infected total knee arthroplasty. *Indian journal of orthopaedics.* 2011;45(6):535-540.

81. Kohl S, Evangelopoulos DS, Kohlhof H, et al. An intraoperatively moulded PMMA prostheses like spacer for two-stage revision of infected total knee arthroplasty. *Knee.* 2011;18(6):464-469.

82. Kusuma SK, Ward J, Jacofsky M, Sporer SM, Della Valle CJ. What is the role of serological testing between stages of two-stage reconstruction of the infected prosthetic knee? *Clin Orthop.* 2011;469(4):1002-1008.

83. Van Thiel GS, Berend KR, Klein GR, Gordon AC, Lombardi AV, Della Valle CJ. Intraoperative molds to create an articulating spacer for the infected knee arthroplasty. *Clin Orthop.* 2011;469(4):994-1001.

84. Borowski M, Kusz D, Wojciechowski P, Cielinski L. Treatment for periprosthetic infection with two-stage revision arthroplasty with a gentamicin loaded spacer. The clinical outcomes. *Ortop.* 2012;14(1):41-54.

85. Cai P, Hu Y, Xie L, Wang L. [Two-stage revision of infected total knee arthroplasty using antibiotic-impregnated articulating cement spacer]. *Chung Kuo Hsiu Fu Chung Chien Wai Ko Tsa Chih.* 2012;26(10):1169-1173.

86. Hardeman F, Londers J, Favril A, Witvrouw E, Bellemans J, Victor J. Predisposing factors which are relevant for the clinical outcome after revision total knee arthroplasty. *Knee Surg Sports Traumatol Arthrosc.* 2012;20(6):1049-1056.

87. Sun Z, Wang L, Sun Y, et al. [Management of deep infection after total knee arthroplasty]. *Chung Kuo Hsiu Fu Chung Chien Wai Ko Tsa Chih.* 2012;26(8):918-921.

88. Lee JK, Choi CH. Two-stage reimplantation in infected total knee arthroplasty using a re-sterilized tibial polyethylene insert and femoral component. *Journal of Arthroplasty.* 2012;27(9):1701-1706.e1701.

89. Lee KJ, Moon JY, Song EK, Lim HA, Seon JK. Minimum Two-year Results of Revision Total Knee Arthroplasty Following Infectious or Non-infectious Causes. *Knee surgery & related research.* 2012;24(4):227-234.

90. Mahmud T, Lyons MC, Naudie DD, Macdonald SJ, McCalden RW. Assessing the gold standard: a review of 253 two-stage revisions for infected TKA. *Clinical Orthopaedics & Related Research.* 2012;470(10):2730-2736.

91. Choi HR, Malchau H, Bedair H. Are prosthetic spacers safe to use in 2-stage treatment for infected total knee arthroplasty? *Journal of Arthroplasty.* 2012;27(8):1474-1479.e1471.

92. Kubista B, Hartzler RU, Wood CM, Osmon DR, Hanssen AD, Lewallen DG. Reinfection after two-stage revision for periprosthetic infection of total knee arthroplasty. *International Orthopaedics.* 2012;36(1):65-71.

93. Jia YT, Zhang Y, Ding C, et al. Antibiotic-loaded articulating cement spacers in two-stage revision for infected total knee arthroplasty: individual antibiotic treatment and early results of 21 cases. *Chinese journal of traumatology = Zhonghua chuang shang za zhi / Chinese Medical Association.* 2012;15(4):212-221.

94. Johnson AJ, Sayeed SA, Naziri Q, Khanuja HS, Mont MA. Minimizing dynamic knee spacer complications in infected revision arthroplasty. *Clin Orthop.* 2012;470(1):220-227.

95. Bruni D, Iacono F, Sharma B, Zaffagnini S, Marcacci M. Tibial tubercle osteotomy or quadriceps snip in two-stage revision for prosthetic knee infection? A randomized prospective study. *Clin Orthop.* 2013;471(4):1305-1318.

96. Joo JH, Lee SC, Ahn NK, Ahn HS, Jung KA. Patellar resurfacing versus no resurfacing in two-stage revision of infected total knee arthroplasty. *Knee.* 2013;20(6):451-456.

97. Kim YS, Bae KC, Cho CH, Lee KJ, Sohn ES, Kim BS. Two-stage revision using a modified articulating spacer in infected total knee arthroplasty. *Knee surgery & related research.* 2013;25(4):180-185.

98. Rajgopal A, Vasdev A, Gupta H, Dahiya V. Revision total knee arthroplasty for septic versus aseptic failure. *J Orthop Surg (Hong Kong).* 2013;21(3):285-289.

99. Mariconda M, Ascione T, Balato G, et al. Sonication of antibiotic-loaded cement spacers in a two-stage revision protocol for infected joint arthroplasty. *BMC musculoskeletal disorders.* 2013;14:193.

100. Silvestre A, Almeida F, Renovell P, Morante E, Lopez R. Revision of infected total knee arthroplasty: two-stage reimplantation using an antibiotic-impregnated static spacer. *Clin.* 2013;5(3):180-187.

101. Zhang Q, Zhou YG, Chen JY, et al. [Treatment of infected total knee arthroplasty with a self-made, antibiotic-loaded cement articulating spacer]. *Zhongguo Gu Shang.* 2013;26(2):119-123.

102. Tigani D, Trisolino G, Fosco M, Ben Ayad R, Costigliola P. Two-stage reimplantation for periprosthetic knee infection: Influence of host health status and infecting microorganism. *Knee.* 2013;20(1):9-18.

103. Castelli CC, Gotti V, Ferrari R. Two-stage treatment of infected total knee arthroplasty: two to thirteen year experience using an articulating preformed spacer. *Int Orthop.* 2014;38(2):405-412.

104. Classen T, von Knoch M, Wernsmann M, Landgraeber S, Loer F, Jager M. Functional interest of an articulating spacer in two-stage infected total knee arthroplasty revision. *Orthop Traumatol Surg Res.* 2014;100(4):409-412.

105. Shaikh AA, Ha CW, Park YG, Park YB. Two-stage approach to primary TKA in infected arthritic knees using intraoperatively molded articulating cement spacers. *Clin Orthop.* 2014;472(7):2201-2207.

106. Pelt CE, Grijalva R, Anderson L, Anderson MB, Erickson J, Peters CL. Two-Stage Revision TKA Is Associated with High Complication and Failure Rates. *Advances in orthopedics.* 2014;2014:659047.

107. Prasad N, Paringe V, Kotwal R, Ghandour A, Morgan Jones R. Two-stage revision for infected total knee arthroplasty: our experience with interval prosthesis. *European journal of orthopaedic surgery & traumatology : orthopedie traumatologie.* 2014;24(7):1279-1283.

108. Puhto AP, Puhto TM, Niinimaki TT, Leppilahti JI, Syrjala HP. Two-stage revision for prosthetic joint infection: outcome and role of reimplantation microbiology in 107 cases. *J Arthroplasty.* 2014;29(6):1101-1104.

109. Sabry FY, Buller L, Ahmed S, Klika AK, Barsoum WK. Preoperative prediction of failure following two-stage revision for knee prosthetic joint infections. *Journal of Arthroplasty.* 2014;29(1):115-121.

**Table A.** Characteristics of Studies Included in Review.

| **Lead Author, Publication Date (Reference No.)** | **Location** | **Year of study** | **Mean /median age (years)** | **% male** | **Follow up**  **Mean/median (months)** | **Type of re-implantation** | **Spacer (Yes/No); articulating or static** | **Spacer type** | **Mean/median interval between stages (months)** | **No. of re-infections** | **No. of participants or knees** | **Quality score** |
| --- | --- | --- | --- | --- | --- | --- | --- | --- | --- | --- | --- | --- |
| **One-stage** |  |  |  |  |  |  |  |  |  |  |  |  |
| Bengtson, 1991 | Sweden | 1986-1989 | NS | NS | NS | NS | NA | NA | NA | 1 | 69 | 9 |
| von Foerster, 1991 | Germany | 1976-1985 | NR | NR | NR | NR | NA | NA | NA | 20 | 118 | 10 |
| Scott, 1993 | UK | 1981-1989 | 71.5 | 20.0 | NS | NS | NA | NA | NA | 2 | 10 | 10 |
| Buechel, 2004 | USA | 1981-1993 | 70.6 | 40.9 | 122.4 | Cemented | NA | NA | NA | 1 | 22 | 12 |
| Sofer, 2005 | Germany | NR | NR | NR | 18.4 |  | NA | NA | NA | 1 | 15 | 11 |
| Bauer, 2006 | France | NS | 71.8 | NS | 52.0 | NS | NA | NA | NA | 4 | 30 | 11 |
| Jenny, 2013 | France | 2004-2007 | 72.0 | 42.6 | 33.0 | Cemented | NA | NA | NA | 6 | 47 | 12 |
| van Kempen, 2013 | Belgium, Netherlands | 2007-2008 | NS | NS | 24.0 | NS | NA | NA | NA | 4 | 34 | 12 |
| Tibrewal, 2014 | UK | 1979-2010 | 66.8 | 34.0 | 126.0 | Cemented and cementless | NA | NA | NA | 3 | 50 | 13 |
| Haddad, 2015 | UK | 2004-2009 | 63.0 | 50.0 | 78.0* | Cemented | NA | NA | NA | 0 | 28 | 13 |
| **Two-stage** |  |  |  |  |  |  |  |  |  |  |  |  |
| Walker, 1984 | USA | 1972-1979 | 66.6 | 71.4 | NS | NS | NS | NS | NS | 2 | 11 | 13 |
| Borden, 1987 | USA | 1977-1984 | NS | NS | 46.0 | Cemented | NS | NS | NS | 1 | 11 | 10 |
| Rosenberg, 1988 | USA | 1981-1986 | 67.0 | 28.0 | 29.0 | NS | NS | NS | NS | 1 | 26 knees | 10 |
| Morrey, 1989 | USA | 1973-1984 | NS | NS | 96.0 | NS | NS | NS | NS | 6 | 15 | 12 |
| Wilson, 1990 | USA | 1973-1987 | NS | NS | 34.0 | NS | NS | NS | NS | 4 | 24 | 10 |
| Windsor, 1990 | USA | 1977-1985 | NS | NS | 48.0 | NS | NS | NS | NS | 1 | 38 knees | 11 |
| Bengtson, 1991 | Sweden | 1986-1989 | NS | NS | NS | NS | NS | NS | 1.00 | 1 | 38 | 9 |
| Henderson, 1991 | USA | 1984-1989 | 67.0 | 35.7 | 27.0 | NS | Yes; Static | NS | 5.30 | 1 | 28 | 13 |
| Whiteside, 1994 | USA | NS | 35-74† | 39.4 | NS | Cementless | Spacer and beads | NS | 1.38 | 4 | 33 | 9 |
| Hanssen, 1994 | USA | 1980-1990 | 68.0 | 47.7 | 52.0 | Cemented and cementless | Spacer and beads | NS | 1.26 | 8 | 89 knees | 10 |
| Masri, 1994 | Canada | 1987-1993 | 66.3 | 52.2 | 26.4 | Cemented | Yes; Articulating | Handmade | 3.12 | 2 | 24 knees | 14 |
| Bose, 1995 | USA | 1983-1991 | 71.0 | 35.3 | 56.4 | Cemented | Beads | NS | NS | 0 | 18 knees | 12 |
| Hofmann, 1995 | USA | 1989-1994 | 70.0 | 53.8 | 30.0 | NS | Yes; Articulating | Handmade | 2.69 | 0 | 26 | 11 |
| Goldman, 1996 | USA | 1977-1993 | 67.0 | 35.0 | 90.0 | NS | Static spacer in 7 knees | NS | 1.84 | 1 | 64 knees | 10 |
| Calton, 1997 | USA | 1987-1996 | 68.0 | 45.8 | 36.0 | Cemented | Yes; Static | Handmade | 1.84 | 2 | 25 knees | 11 |
| Gacon, 1997 | France | 1984-1994 | 75.5 | 31.0 | 42.0 | Cemented | Yes; Static | NS | 1.93 | 5 | 29 | 10 |
| McPherson, 1997 | USA | 1993-1996 | NS | 38.1 | 16.8 | Cemented | NS | NS | 5.75 | 1 | 21 | 11 |
| Hirakawa, 1998 | USA | 1980-1993 | 67.0 | 46.3 | 61.9 | Cemented | Yes; NS | NS | 1.38 | 12 | 55 knees | 10 |
| Jerosch, 1999 | Germany | NS | NS | NS | NS | NS | NS | NS | NS | 4 | 10 | 9 |
| Lecuire, 1999 | France | 1989 onwards | 70.5 | NS | 16.5 | Cemented and cementless | No | NA | 1.38 | 0 | 12 | 10 |
| Segawa, 1999 | USA | 1980-1995 | 67.0* | NS | 39.6 | NS | Spacer and beads | NS | 1.81 | 5 | 29 infections | 13 |
| Barrack, 2000 | USA, Canada | NS | 68.5 | NS | NS | NS | Yes; Static | NS | 1.0-1.4 | 2 | 28 | 14 |
| Fehring, 2000 | USA | 1986-1999 | NS | NS | 36.0 | NS | Yes; Static | NS | NS | 3 | 25 | 11 |
| Fehring, 2000 | USA | 1999 | NS | NS | 27.0 | NS | Yes; Articulating | Handmade | NS | 1 | 30 | 11 |
| Haddad, 2000 | Canada | 1987-1996 | 69.0 | 42.2 | 48.0 | Cemented | Yes; Articulating | Commercial and handmade | 3.56 | 4 | 45 | 13 |
| Mont, 2000 | USA | 1989-1993 | 64.0 | 48.6 | 68.0 | Cemented | Yes; Static | NS | NS | 5 | 35 | 14 |
| Lonner, 2001 | USA | 1983-1997 | NS | NS | 56.0 | Cemented | Yes; Static | NS | NS | 6 | 56 knees | 11 |
| Emerson, 2002 | USA | 1986-1994 | 65.7 | 57.1 | 90.0 | NS | Yes; Static | NS | NS | 1 | 26 | 13 |
| Emerson, 2002 | USA | 1995-1999 | 65.1 | 52.9 | 45.6 | NS | Yes; Articulating | NS | NS | 0 | 22 | 13 |
| Husted, 2002 | Denmark | 1989-2000 | 73.2 | 17.6 | NS | Cemented | NS | Commercial | NS | 2 | 17 | 10 |
| Siebel, 2002 | Germany | NS | 66.1 | 30.0 | 18.1 | Cemented | NS | Handmade | 1.89 | 0 | 10 | 11 |
| Pietsch, 2003 | Austria | NS | NS | NS | 14.8 | NS | Yes; Articulating | NS | 16.00 | 1 | 24 | 12 |
| Evans, 2004 | USA | 1995-2002 | NS | NS | 24.0*** | NS | Yes; Articulating | Handmade | NS | 2 | 31 | 12 |
| Haleem, 2004 | USA | 1989-1994 | 69.0 | 53.2 | 86.4 | Cemented | Yes; Static | NS | 1.40 | 4 | 96 knees | 11 |
| Meek, 2004 | Canada | 1997-1999 | NS | 42.6 | 41.0* | NS | Yes; Articulating | NS | NS | 2 | 54 | 11 |
| Durbhakula, 2004 | USA | 1998-2001 | 72.0 | 41.7 | 33.0 | Cemented | Yes; Articulating | Handmade | 2.80 | 2 | 24 | 13 |
| Cuckler, 2005 | USA | 1994-2002 | 68.0 | 25.0 | 64.8 | NS | Yes; Articulating | NS | NS | 1 | 44 | 10 |
| Pitto, 2005 | New Zealand | 2000-2003 | 67.0 | 42.9 | 24.0 | Cemented | Yes; Articulating | Handmade | 2.80 | 0 | 21 | 14 |
| Musil, 2005 | Czech Republic | 1990-2003 | NS | NS | 34.5 | NS | NS | NS | 3.54 | 2 | 10 | 11 |
| Hoad-Reddick, 2005 | UK | 1992-2001 | 70.6 | 54.2 | 56.4 | NS | Yes; Static | Handmade | NS | 4 | 53 | 14 |
| Hofmann, 2005 | USA | 1989-2001 | 67.0 | 50.0 | 73.0 | Cemented | Yes; Articulating | Handmade | 2.80 | 2 | 50 | 13 |
| Bauer, 2006 | France | NS | 68.3 | NS | 52.0 | NS | NS | NS | NS | 12 | 77 | 11 |
| Huang, 2006 | Taiwan | 1996-2002 | 68.7 | 26.3 | 52.2 | Cemented | Yes; Articulating | NS | 4.76 | 1 | 21 knees | 13 |
| Hart, 2006 | UK | 1998-2003 | 68.2 | 58.3 | 48.5 | Cemented | Yes; Articulating | Handmade | 4.30 | 6 | 48 | 12 |
| Jamsen, 2006 | Finland | 1993-2003 | 68.0 | 34.4 | 32.0 | Cemented | Yes; Articulating and static | NS | 5.20 | 2 | 34 | 12 |
| Souillac, 2006 | France | 2000-2003 | NS | NS | 28.0 |  | Yes; Articulating | Handmade | 2.00 | 4 | 28 | 11 |
| Trezies, 2006 | Australia | 1992-2004 | 66.6 | 63.6 |  | NS | Yes; Articulating | NS | NS | 1 | 11 | 9 |
| Laffer, 2006 | Switzerland | 1988-2003 | 70.0 | NS | 28.0 | NS | Yes (In 5 patients); NS | NS | NS | 2 | 13 | 11 |
| Cordero-Ampuero, 2007 | Spain | 1996-2003 | 72.8 | NS | NS | Cemented | Yes; Static | Handmade | 10.0 | 1 | 24 | 14 |
| Freeman, 2007 | USA | 1986-2004 | 64.9 | NS | 62.2 | NS | Yes; Articulating | Handmade | NS | 4 | 76 knees | 10 |
| Freeman, 2007 | USA | 1986-2004 | 71.2 | NS | 86.6 | NS | Yes; Static | Handmade | NS | 3 | 38 knees | 10 |
| Hsu, 2007 | China | 1998-2004 | NS | NS | 58.0 | NS | Yes; Articulating | Handmade | 3.20 | 2 | 21 | 12 |
| Pascale, 2007 | Italy | 2005-2006 | 68.0 | 21.4 | 12.0 | NS | Yes; Articulating | Handmade | 2.07 | 0 | 14 | 10 |
| Thabe, 2007 | Germany | 1992 onwards | 72.3 | NS | NS | Cemented | NS | Handmade | 0.90 | 0 | 20 | 12 |
| Babis, 2008 | Greece | 1995-2005 | 71.0 | 25.0 | 72.0 | NS | Yes; Articulating | Handmade | 1.38 | 0 | 24 | 13 |
| Hsu, 2008 | Taiwan | 1991-2001 | 66.0 | 25.8 | 68.3 | Cemented stems | Yes; Static | NS | NS | 4 | 32 knees | 12 |
| Villanueva-Martinez, 2008 | Spain | NS | 71.0 | 23.3 | NS | Cemented | Yes; Articulating | Handmade | 3.20 | 1 | 30 | 13 |
| Anderson, 2009 | USA | 1997-2004 | 64.0 | 40.0 | 54.0 | Cemented | Yes; Articulating | NS | 2.53 | 1 | 25 | 14 |
| Ghanem, 2009 | USA | 1999-2006 | 68.0 | 51.4 | 33.6 | Cemented | NS | NS | 3.52 | 23 | 109 | 12 |
| Peters, 2009 | USA | 1995-2007 | NS | NS | 49.0 | NS | Yes; Articulating | Commercial | 3.00 | 9 | 53 knees | 12 |
| Pietsch, 2009 | Austria | 1999-2002 | NS | NS | 47.0 | NS | Yes; Articulating | Handmade | NS | 3 | 33 | 13 |
| Su, 2009 | Taiwan | 2003-2005 | 72.0 | 33.3 | 47.5 | Cemented | Yes; Articulating | Handmade | 3.50 | 1 | 15 | 13 |
| Cordero-Ampuero, 2010 | Spain | 1997-2008 | 72.0 | 27.7 | 57.6 | Cemented | Yes; Static | Handmade | NS | 4 | 47 | 13 |
| Ocguder, 2010 | Turkey | 2003-2006 | 63.0** | 41.2 | 20.0 | Cemented | Yes; Articulating | Commercial | 4.20 | 2 | 17 | 12 |
| Patil, 2010 | USA | 1999-2006 | NS | NS | 40.0 | Cemented | Yes; Static | NS | 5.40 | 1 | 14 | 12 |
| Kurd, 2010 | USA | 1998-2005 | 67.0** | 54.9 | 35.0 | Cemented | Yes; Static | NS | 3.45 | 26 | 102 | 11 |
| Mortazavi, 2010 | USA | 1998-2005 | 66.0 | 37.2 | 65.0 | NS | NS | NS | 13.2 | 44 | 475 | 11 |
| Shen, 2010 | China | 2004-2007 | 67.0 | 41.2 | 31.0 | Cemented | Yes; Articulating | NS | 7.80 | 1 | 17 | 11 |
| Park, 2010 | South Korea | 2000-2005 | 66.5 | 10.0 | 36.0 | NS | Yes; Static | Handmade | NS | 3 | 20 | 12 |
| Park, 2010 | South Korea | 2003-2007 | 60.2 | 12.5 | 29.0 | NS | Yes; Articulating | Handmade | NS | 1 | 16 | 12 |
| Ritter, 2010 | USA | 1969-2004 | 65.0 | NS | 63.6 | NS | NS | NS | NS | 17 | 51 | 12 |
| Westrich, 2010 | USA | 1998-2006 | 66.1 | 51.6 | 52.4 | Cemented | Yes; Articulating and static | Handmade | NS | 20 | 99 knees | 12 |
| Cabo, 2011 | Spain | 2004-2009 | 70.0 | 10.0 | 24.0 | Cementless | Yes; Static | Handmade | 4.10 | 3 | 25 | 13 |
| Choi, 2011 | USA | 2002-2007 | 66.6 | 43.8 | 36.0 | NS | Yes; Articulating and static | NS | 4.40 | 6 | 32 | 12 |
| Ferrari, 2011 | Italy | 2000 onwards | NS | NS | NS | NS | Yes; Articulating | Commercial | 2.76 | 4 | 50 | 11 |
| Garg, 2011 | India | 2002-2007 | 62.0 | 22.2 | 62.0 | NS | Yes; Articulating | Handmade | NS | 0 | 36 | 12 |
| Kohl, 2011 | Switzerland | 2006-2007 | 73.1 | 75.0 | NS | Cemented | NS | Handmade | 3.50 | 0 | 16 | 12 |
| Kusuma, 2011 | USA | NS | 65.5 | 45.0 | NS | NS | Yes; NS | NS | 2.43 | 8 | 76 | 10 |
| Van Thiel, 2011 | USA | 2005-2007 | 66.0 | 51.7 | 35.0 | NS | Yes; Articulating | Handmade | 2.46 | 6 | 60 | 12 |
| Borowski, 2012 | Poland | 2007-2010 |  |  |  |  |  | Commercial |  | 2 | 12 | 11 |
| Cai, 2012 | China | 2007-2009 | 65.2 | 65.2 | 43.2 | NS | Yes; Articulating | NS | NS | 2 | 23 | 11 |
| Hardeman, 2012 | Belgium | 1995-2008 | 64.4 | 38.1 | 66.0 | Cemented | Yes; Articulating and static | Handmade | 2.2 | 4 | 21 | 11 |
| Sun, 2012 | China | 2004-2010 | NS | NS | NS | NS | NS | NS | NS | 3 | 12 | 11 |
| Lee, 2012 | South Korea | 2000-2006 | 64.8 | 10.5 | 67.0 | Cemented | Yes; Articulating | Handmade | 5.00 | 1 | 20 knees | 11 |
| Lee, K-J, 2012 | South Korea | 2004-2009 | 68.3 | 14.3 | 40.2 | Cemented | Yes; Articulating | Handmade | 7.30 | 3 | 21 | 11 |
| Mahmud, 2012 | Canada | 1993-2010 | 70.0 | 43.5 | 48.0 | NS | Yes; Articulating and static | NS | NS | 16 | 253 knees | 12 |
| Choi, 2012 | USA | 2000-2009 | 64.0 | 48.9 | 58.0 |  | Yes; Articulating | NS | 6.00 | 4 | 14 | 11 |
| Choi, 2012 | USA | 2000-2009 | 64.0 | 48.9 | 58.0 |  | Yes; Static | Handmade | 6.00 | 11 | 33 | 11 |
| Kubista, 2012 | USA | 1998-2006 | NS | NS | 93.6 | NS | NS | NS | NS | 58 | 368 | 11 |
| Jia, 2012 | China | 2006-2009 | NS | NS | 32.2 | NS | Yes; Articulating | Handmade | 2.64 | 0 | 21 | 11 |
| Johnson, 2012 | USA | 2000-2009 | 62 | NS | 27.0 | NS | Yes; Articulating | Commercial | 3.06 | 6 | 34 | 11 |
| Johnson, 2012 | USA | 2000-2009 | 61 | NS | 66.0 | NS | Yes; Static | Handmade | 3.52 | 14 | 81 | 11 |
| Bruni, 2013 | Italy | 1997-2004 | 73.0 | 24.7 | 144.0 | NS | Yes; Static | Handmade | NS | 5 | 90 | 15 |
| Joo, 2013 | South Korea | 2007-2008 | 68.3 | 6.3 | 28.8 | NS | Yes; Static | NS | 1.54 | 2 | 49 knees | 12 |
| Kim, 2013 | South Korea | 2006-2011 | 61.5 | 25.0 | 22.3 | NS | Yes; Articulating | NS | 3.30 | 2 | 20 | 12 |
| Rajgopal, 2013 | India | 2000-2008 | NS | 35.4 | 71.6 | NS | Yes; Articulating | NS | 2.07 | 5 | 67 knees | 12 |
| Mariconda, 2013 | Italy | 2009-2011 | 67.3 | 56.3 | 28.9 | NS | NS | Handmade | 3.90 | 1 | 16 | 13 |
| Silvestre, 2013 | Spain | 2000-2007 | 72.0 | 31.1 | 86.0 | NS | Yes; Static | NS | 4.40 | 2 | 48 knees | 10 |
| Zhang, 2013 | China | 2002-2007 | 59.6 | 45.5 | 34.5 | NS | Yes; Articulating | Handmade | 4.70 | 0 | 22 | 12 |
| Tigani, 2013 | Italy | 2000-2006 | 68.0 | 32.4 | 65.0 | Cementless | Yes; Articulating | Handmade | NS | 8 | 38 knees | 13 |
| Castelli, 2014 | Italy | 2000 onwards | 68.0 | 36.0 | 84.0 | NS | Yes; Articulating | Commercial | 3.68 | 4 | 50 | 12 |
| Classen, 2014 | Germany | 2003-2008 | 71.0 | 26.1 | 47.0 | Cemented | Yes; Articulating | Handmade | 6.05 | 3 | 23 | 13 |
| Shaikh, 2014 | South Korea | 2001-2009 | 65.0 | 38.5 | 48.0 | Cemented | Yes; Articulating | Handmade | 5.60 | 0 | 15 | 11 |
| Pelt, 2014 | USA | 1998-2010 | 63.0 | 59.0 | 38.0 | NS | NS | NS |  | 21 | 58 knees | 11 |
| Prasad, 2014 | UK | 2001-2008 | 66.0 | 53.3 | 60.0 | NS | NS | NS | 5.98 | 4 | 60 | 12 |
| Puhto, 2014 | Finland | 2001-2009 |  |  |  |  |  | Commercial |  | 5 | 46 | 11 |
| Sabry, 2014 | USA | 1996-2010 | 65.0 | 52.5 | 39.9 | NS | NS | NS | 3.38 | 105 | 314 | 13 |
| Haddad, 2015 | UK | 2004-2009 | 68.0 | 44.6 | 78.0 | Cemented | Yes; Articulating | Handmade | 2.04 | 5 | 74 | 13 |

NA, not applicable; NR, not retrieved; NS, not stated; *, relates to whole group; **, mean age at reimplantation; ***, minimum followup; †, age range; Both, some participants had cemented re-implantation and others cementless

**Figure A**. Rates of re-infection in patients treated by one-stage revision, grouped according to study and population level characteristics

CI, confidence interval (bars); *, *P*-value for meta-regression; †, number of infections and participants do not add up to the overall total because of missing data

**Figure B**. Rates of re-infection in patients treated by two-stage revision, grouped according to study and population level characteristics

CI, confidence interval (bars); *, *P*-value for meta-regression; †, number of infections and participants do not add up to the overall total because of missing data

**Figure C**. Filled funnel plot with 95% confidence limits after trim-and-fill method
